# Impact of Autophagy on Gene Expression and Tapetal Programmed Cell Death During Pollen Development in Rice

**DOI:** 10.3389/fpls.2020.00172

**Published:** 2020-03-06

**Authors:** Shigeru Hanamata, Jumpei Sawada, Seijiro Ono, Kazunori Ogawa, Togo Fukunaga, Ken–Ichi Nonomura, Seisuke Kimura, Takamitsu Kurusu, Kazuyuki Kuchitsu

**Affiliations:** ^1^ Department of Applied Biological Science, Tokyo University of Science, Noda, Japan; ^2^ Imaging Frontier Center, Tokyo University of Science, Noda, Japan; ^3^ Graduate School of Science and Technology, Niigata University, Niigata, Japan; ^4^ Plant Cytogenetics Laboratory, National Institute of Genetics, Mishima, Japan; ^5^ Faculty of Life Sciences, Kyoto Sangyo University, Kyoto, Japan; ^6^ Center for Ecological Evolutionary Developmental Biology, Kyoto Sangyo University, Kyoto, Japan; ^7^ Department of Mechanical and Electrical Engineering, Suwa University of Science, Chino, Japan

**Keywords:** autophagy, rice tapetum, programmed cell death, gene regulatory networks, quality control

## Abstract

Autophagy has recently been shown to be required for tapetal programmed cell death (PCD) and pollen maturation in rice. A transcriptional regulatory network is also known to play a key role in the progression of tapetal PCD. However, the relationship between the gene regulatory network and autophagy in rice anther development is mostly unknown. Here, we comprehensively analyzed the effect of autophagy disruption on gene expression profile during the tapetal PCD in rice anther development using high-throughput RNA sequencing. Expression of thousands of genes, including specific transcription factors and several proteases required for tapetal degradation, fluctuated synchronously at specific stages during tapetal PCD progression in the wild-type anthers, while this fluctuation showed significant delay in the autophagy-deficient mutant *Osatg7-1*. Moreover, gene ontology enrichment analysis in combination with self-organizing map clustering as well as pathway analysis revealed that the expression patterns of a variety of organelle-related genes as well as genes involved in carbohydrate/lipid metabolism were affected in the *Osatg7-1* mutant during pollen maturation. These results suggest that autophagy is required for proper regulation of gene expression and quality control of organelles and timely progression of tapetal PCD during rice pollen development.

## Introduction

Reproductive development, both in animals and plants, requires drastic metabolic changes for nutrient supply to gametes. In flowering plants, anthers exhibit a four-layered structure composed of epidermis, endothecium, middle layer, and tapetum. The tapetum, the innermost of the four sporophytic layers of the anther wall, directly contacts with the developing microspores and provides metabolites and nutrients to pollen grains and pollen coat during their development (Ariizumi and Toriyama, 2011). Therefore, proper metabolic regulation is critical for proper pollen maturation and grain yield.

The tapetum acts as a nutritional source for the developing microspores by undergoing degeneration triggered by programmed cell death (PCD) from stage 7 (ST7) to ST11 (Ariizumi and Toriyama, 2011; Zhang and Yang, 2014). After two rounds of cell division during meiosis, tetrads covered by callose are formed at ST8. After callose degradation, at ST9, haploid microspores are released into the lobe, and the accumulation of starch and lipids are associated with the pollen maturation and pollen wall development (Zhang et al., 2008; Zhang et al., 2011).

Transcriptional regulatory network plays key roles in the progression of anther/pollen development in plants. Transcriptomic analyses of developing anthers and pollen grains have been described in many species (Huang et al., 2011; Rutley and Twell, 2015) and enriched our knowledge of the repertoire of genes expressed during anther/pollen development. Transcriptomic profiling at different developmental stages has been reported for *Arabidopsis* (Honys and Twell, 2004), rice (Wei et al., 2010), and tobacco (Bokvaj et al., 2015).

Several key transcription factors (TFs) required for anther and pollen development as well as tapetal PCD have been identified by forward genetic screens in *Arabidopsis*. A basic helix-loop-helix (bHLH) transcription factor (TF) *DYSFUNCTIONAL TAPETUM1* (*DYT1*) plays a critical role in regulating tapetum function and pollen development (Zhang et al., 2006; Feng et al., 2012; Zhu et al., 2015). DYT1 regulates the expression of genes preferentially expressed in the tapetum such as *ABORTED MICROSPORES* (*AMS*) (Sorensen et al., 2003; Xu et al., 2014) and *MALE STERILITY1* (*MS1*) (Vizcay-Barrena and Wilson, 2006), primarily *via DEFECTIVE IN TAPETAL DEVELOPMENT AND FUNCTION1* (*TDF1*) (Gu et al., 2014). *AtMYB103* participates in the tetrad callose degradation (Higginson et al., 2003; Zhang et al., 2007).

Rice is not only a widely-grown crop to feed almost half of the global population but also an ideal monocot model plant facilitating plant science research, *e.g.* the discovery of mechanisms underlying male gametophyte development (Kim and Zhang, 2018), and thus is of significance for both basic and applied plant research. Several bHLH-TFs including *undeveloped tapetum1* (*UDT1*) (Jung et al., 2005), *tapetum degeneration retardation* (*TDR*) (Li et al., 2006), *TDR-interacting protein2* (*TIP2*) (Fu et al., 2014; Ko et al., 2014), and *eternal tapetum1* (*EAT1*) (Niu et al., 2013; Ono et al., 2018), a MYB transcription factor *GAMYB* (Aya et al., 2009), and a plant homeodomain (PHD)-finger motif DNA binding protein *persistent tapetal cell1* (*PTC1*) (Li et al., 2011) have been reported to be required for tapetal PCD and identified as key genes involved in a transcriptional network during anther development as well as tapetal PCD progression. Comparative studies with *Arabidopsis* developing anthers including tapetum have revealed complexed and different expression pattern of genes during reproductive periods (Li et al., 2006; Deveshwar et al., 2011).

Autophagy is a major intracellular mechanism that degrades organelles, proteins, and metabolites (Mizushima and Komatsu, 2011). The double membrane structure called autophagosomes is formed in the cytosol, which fuses with the lytic compartments, the vacuoles or lysosomes, where the components enclosed in the autophagosomes are degraded (Kurusu et al., 2016). More than 30 autophagy-related genes (*ATGs*) required for autophagy are well conserved basically in all eukaryotes including animals and plants (Avin-Wittenberg et al., 2012; Yoshimoto and Ohsumi, 2018).

Autophagy plays essential roles in growth, development, and survival in eukaryotic cells (Chung et al., 2009; Li and Vierstra, 2012; Yoshimoto, 2012). Under normal growth conditions, autophagy plays critical roles in nutrient recycling, cell waste management and quality control of organelles as a housekeeping mechanism. When cells are faced with nutrient deprivation or stress, remobilization of nutrients by autophagy becomes crucial (Thompson et al., 2005; Shibata et al., 2013; Yoshimoto et al., 2014).

In plants, autophagy has been suggested to play roles in the recycling of proteins and metabolites, including lipids, at the whole-plant level, and is involved in numerous physiological processes (Ghiglione et al., 2008; Ishida et al., 2008; Chung et al., 2009; Yoshimoto et al., 2009; Kwon et al., 2010; Izumi et al., 2015; Izumi et al., 2017). Transcriptomics and metabolomics in autophagy-deficient mutants of *Arabidopsis* (*atatg5-1*) and *maize* (*Zmatg12*) suggest the importance of autophagy in cell homeostasis and stress responses. These multi-omics also provides comprehensive data sets for the identification of proteins, protein complexes, organelles and processes directly or indirectly under autophagic control.

In rice, autophagy contributes to degradation of chloroplasts/plastids (Izumi et al., 2015) and efficient nitrogen remobilization and biomass production at the whole-plant level by facilitating protein degradation for nitrogen recycling (Wada et al., 2015) as well as starch metabolism during seed development (Sera et al., 2019). Rice mutants defective in autophagy, *Osatg7-1*, *Osatg7-2*, and *Osatg9*, show sporophytic severe male sterility under normal growth conditions (Kurusu et al., 2014). Autophagy is involved in phytohormone and lipid metabolism in anthers and is crucial for sexual reproductive development (Kurusu et al., 2014; Kurusu et al., 2017; Kurusu and Kuchitsu, 2017). Pollens from the *Osatg7-1* mutant are premature due to significant defects in anthers during pollen maturation (Kurusu et al., 2014), and autophagy is induced inside the tapetum at the uninucleate stage (ST9–10) (Hanamata et al., 2019). Moreover, transmission electron microscopy (TEM) analysis has shown that the morphology of tapetum at ST8–9 is normal, but the tapetal collapse is significantly delayed and intracellular structures including mitochondria and plastids remained at mature pollen stage in the autophagy-deficient mutant *Osatg7-1* (Hanamata et al., 2014; Kurusu et al., 2014), suggesting that autophagy contributes to tapetal degradation and PCD in rice. However, the relationship between gene regulatory network and autophagy in plant anther development and tapetal PCD progression is mostly unknown.

In this report, to reveal the role of autophagy and how autophagy affects the gene regulatory network during anther/pollen development and tapetal PCD, we performed RNA-sequencing (RNA-seq)-based transcriptome analyses in combination with a quantitative PCR (qPCR) of anthers from ST8 to ST11 of the wild-type (WT) and the autophagy-deficient mutant *Osatg7-1* plants. Role of autophagy in the synchronized progression of tapetal PCD, metabolisms of carbohydrates and lipids and the quality control of organelles during rice anther/pollen development are discussed.

## Materials and Methods

### Plant Materials and Sample Preparation of Anthers

Surface-sterilized seeds of transgenic rice lines (*Oryza sativa* L. cv. *Nipponbare* (NB)) were germinated on MS medium (Murashige and Skoog, 1962) containing 0.8% agar and grown for 10 days in a growth chamber in long-day conditions (16-h light/8-h darkness, 28°C). Seedlings were transplanted into soil and grown in a greenhouse or paddy field at the National Institute of Genetics (NIG) in Mishima (Japan).

*Tos17*-insertional rice *Osatg7-1* mutant (*OsATG7−/−*), wild-type (*OsATG7+/+*), and heterozygous (*OsATG7+/−*) plants were selected in seed pools obtained from heterozygous plants by genomic PCR using the following primers: *OsATG7* forward primer 5′-CATACTACCACCTCAGCTTGCTAG-3′, *Tos-17* forward primer 5′-ACTATTGTTAGGTTGCAAGTTAGTTAAGA-3′, and *OsATG7* reverse primer 5′-GCATTCAGGAAAACCTCGTATCG-3′. The original parental cultivars of the *Tos17*-insertional mutant and NB were also used as control plants for *Osatg7-1*.

### Sample Preparation for RNA Extraction

Anther samples at different developmental stages were separated based on the length and color of the anthers (**Table 1**), immediately frozen with liquid nitrogen in microtubes, and stored at −80°C. More than 180 anthers from 30 flowers were used at each stage sample.

**Table 1:** Anther groups defined for RNA-seq analysis.

Pollen developmental stages	Glumous flower			Anther	
	Length (mm)	Colors	Textures	Anther length (mm)	Colors
Tetrad stage (ST8)	3.5–4.5	White	Soft	0.8–0.9	Transparent
Early uninucleate stage (ST9)	4.5–5.2	White	Soft	0.9–1.0	Transparent
Late uninucleate stage early (ST10E)	5.2–5.8	Whitish green	Soft	1.1–1.2	Transparent
Late uninucleate stage late (ST10L)	5.8–6.2	Whitish green	Hard	1.3–1.5	Whitish yellow
Bicellular stage (ST11)	6.2-	Green	Hard	1.6–1.8	Yellow

Total RNA was extracted from stage 8 to stage 11 rice anthers, with three biological replicates each, using TRIzol reagent in accordance with the manufacturer's instructions (Life Technologies) and treated with DNase I (TaKaRa). Quality and integrity of RNA were checked using a spectrophotometer and Agilent 2100 bioanalyzer (Agilent).

### RNA-sequencing and Gene Expression Profiling

RNA-seq libraries were prepared using the Illumina TruSeq^®^ Stranded RNA LT kit (Illumina), according to manufacturer instructions. To find differentially expressed genes (DEGs) throughout developmental stages in WT as well as *Osatg7-1* mutant anthers, 30 libraries were prepared and sequenced using the NextSeq500 sequencing platform (Illumina) in accordance with the manufacturer's instructions. Approximately 20 million raw reads giving more than 3 Gb sequence data for each sample were obtained by single-end sequencing of 75 bp length. The data were deposited to the DDBJ Sequencing Read Archive database (Accession number DRA008977). The obtained reads were mapped to the reference rice genome (IRGSP-1.0) by TopHat2 (Kim et al., 2013), and the htseq-counts script in the HTSeq library was used to count the reads (Anders et al., 2015). Count data were subjected to trimmed mean of M-values (TMM) normalization in EdgeR (Robinson et al., 2010; McCarthy et al., 2012). Multidimensional scaling was performed *via* calculating log-fold changes between WT and using DEGs to compute distances in EdgeR with the “plotMDS” function (**Supplementary Figure S1**).

Transcript expression profiles and DEGs were defined using EdgeR generalized linear models (GLMs) (Robinson et al., 2010). Differential expression was calculated *via* fitting a GLM at the gene level using either developmental stages (from ST8 to ST11) or autophagy dependence as factors. The threshold for DEGs was a false discovery rate (FDR) of  < 0.01; this yielded 20,391 genes. As a result, RAP-ID accessions were assigned in 19,748 genes of all DEGs, and these 20,391 genes were designated as anther-specific DEGs (ASDs). Moreover, for defining all DEGs under the control of autophagy activation during anther development, differential expression was calculated *via* fitting a GLM at the gene level using autophagy dependence as a factor. As a result, 2,359 genes were designated as autophagy-dependent DEGs (ADDs).

### Bioinformatic Analyses: Principal Components Analysis with Self-Organizing Map Clustering and Gene Ontology Analysis

We applied a gene-expression clustering method (Chitwood et al., 2013) for both ASDs and ADDs defined using EdgeR. Scaled expression values were used for multilevel 3 × 3 and 4 × 4 rectangular self-organizing map (SOM) clusters (**Figures 2** and **7**) (Kohonen, 1982; Wehrens and Buydens, 2007). One hundred training interactions were used during clustering. Gene clusters were based on the final assignment of genes to winning units. In order to focus on gene-expression patterns instead of expression magnitude and to identify genes that vary in expression patterns between the WT and *Osatg7-1*, expression values were mean-centered and variance-scaled separately between the WT and *Osatg7-1* in a 3 × 3 rectangular SOM.

Principal component analysis (PCA) was performed with PC values calculated from gene expression across samples (R stats package, prcomp function). For 4 × 4 rectangular SOM clusters, network graphics in Gephi (Bastian et al., 2009) were used to visualize—as a directed network—the assignment of genes from different accessions to separate clusters. Direction of arrows indicates gene assignment to clusters, from the WT to *Osatg7-1*, with arrow size proportional to the gene number represented. Clustered and displaced gene sets among clusters were subjected to gene ontology (GO) analysis using agriGO v2.0 (http://systemsbiology.cau.edu.cn/agriGOv2/).

The MapMan program allows the grouping of genes into different functional categories and visualization of data through various diagrams (Jung and An, 2012). To obtain functional classifications, we uploaded RAP locus IDs for 1,556 ADDs [early phase of stage 10 (ST10E); FDR < 0.05] to the MapMan tool kit. We then investigated the overviews of both regulation and metabolism pathways containing photosynthesis pathways, carbohydrate metabolism, *N*-dependent pathways such as amino acid, cell wall, lipid, and secondary metabolisms.

### Quantification of mRNA by Real-Time PCR

First-strand complementary DNA (cDNA) was synthesized from 500 ng of total RNA with ReverTra Ace^®^ qPCR RT Master Mix with gDNA Remover (TOYOBO). Real-time PCR was performed using a Bio-Rad CFX Connect Real-Time System (Bio-Rad) with the THUNDERBIRD^®^ SYBR qPCR Mix (TOYOBO) and the specific primers (**Supplementary Table S1**). Relative mRNA levels were calculated using the 2^-ΔΔCt^ method and normalized to corresponding an *OsUbiquitin5* gene (Os01g0328400) level. The relative level of each gene in the WT anthers at tetrad stage (ST8) was standardized as 1.

## Results and Discussion

### Transcriptomic Analyses by RNA Sequencing

**Figure 1:**
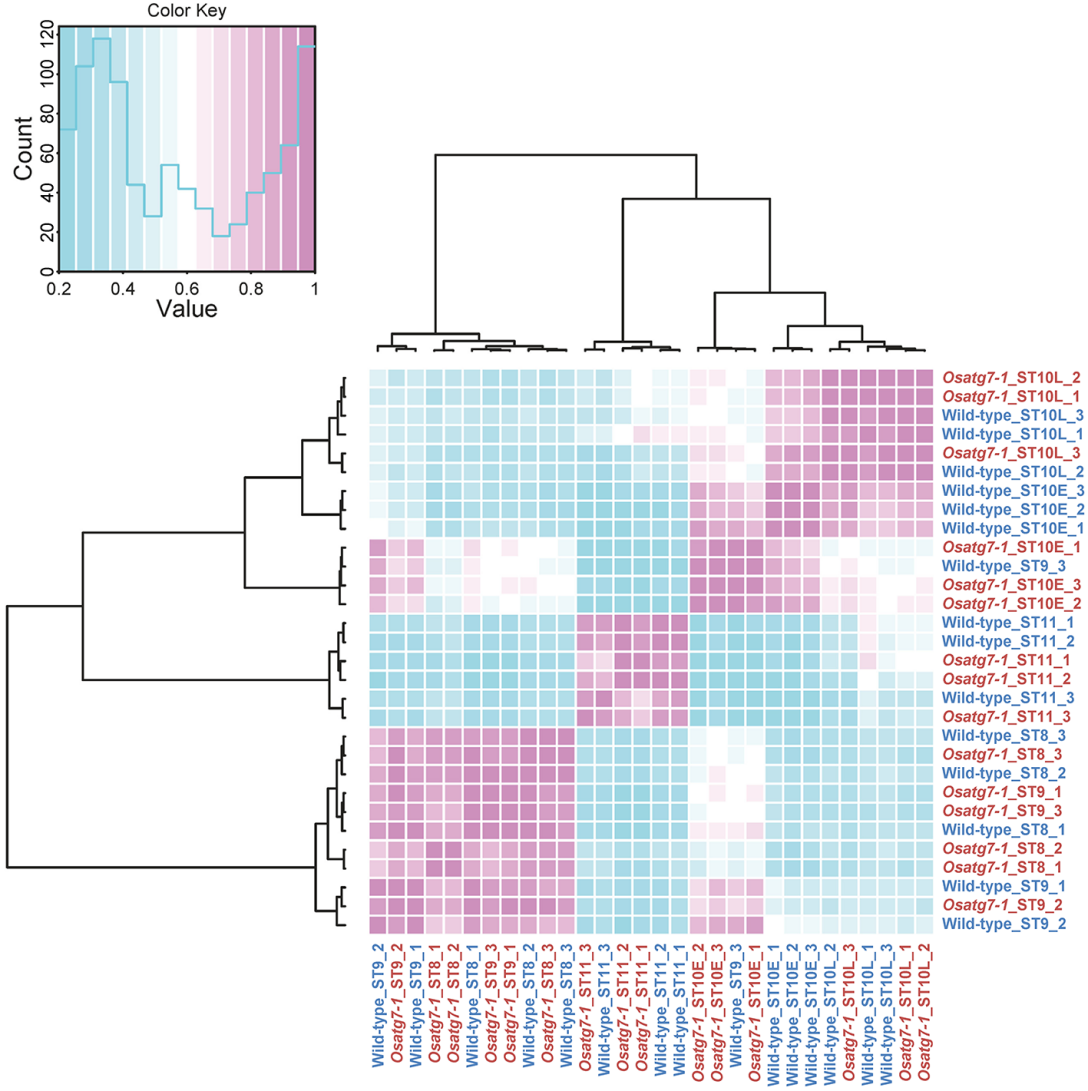
Correlation matrix analyses of gene expressions in rice anthers. Correlation matrix of all 30 RNA-seq samples of the WT and *Osatg7-1* anthers throughout developmental stages. The analyses were performed by comparing the values of the entire transcriptome in all 30 samples with three biological replicates. Correlation analyses were performed using the R software.

Autophagy has been shown to be induced at the uninucleate stage throughout the tapetal cells during anther development (Kurusu et al., 2014; Hanamata et al., 2019). To identify genes affected by the activation of autophagy throughout the reproductive period, we conducted RNA-seq experiments using whole anther samples and compared the data between WT and *Osatg7-1* plants. We obtained data from five different stages: tetrad (ST8), early uninucleate stage (ST9), late uninucleate stages (early and late phases of stage 10, ST10E and ST10L, respectively), and bicellular stage (ST11), each with three biological replicates (**Table 1**). Overall, 210,249,723 reads from the WT and 212,842,580 reads from the *Osatg7-1* anthers were mapped to the rice genome. The reliability between each sample was confirmed using multidimensional scaling (MDS) plot analysis (**Supplementary Figure S1**). To confirm the trends in gene expression levels of anther samples from different stages between the WT and the *Osatg7-1* mutant, we used correlation matrix (**Figure 1**). Similar expression patterns mainly depended on the developmental stage rather than on autophagy disruption. This trend was also confirmed by MDS plot analysis (**Supplementary Figure S1**).

### Anther-Specific Differentially Expressed Genes and Induction of Autophagy- Related Genes During Pollen Maturation

To define all differentially-expressed genes (DEGs) throughout developmental stages in the WT and the *Osatg7-1* mutant anthers, we used EdgeR package in R software. Of 38,311 genes located on the rice genome, 20,391 genes were defined as anther-specific DEGs (ASDs) based on a GLM at the gene level using either developmental stages (from ST8 to ST11) or autophagy dependence as factors (FDR < 0.01) (**Supplementary Dataset 1**). The lists of ASDs that were significantly up- or down-regulated in each developmental stage are shown in **Supplementary Dataset 1**.

We found that the expression patterns of a variety of *ATG* genes including *ATG8s* defined as ASDs were up-regulated from ST10E to ST11 in the WT throughout anther development (**Supplementary Figure S2**). Ectopic overexpression of *OsATG8s*, such as *ATG8a* and *ATG8b*, has been shown to result in enhancement of autophagic activity in rice (Yu et al., 2019; Zhen et al., 2019). Since autophagy is dramatically induced at the uninucleate stages (ST9–10) throughout the tapetal cells during pollen maturation (Hanamata et al., 2019), sustained-autophagic activity in rice tapetum may be regulated by transcription of *ATG* genes including *ATG8s* at least in part.

### Visualization and Assessment of Self-Organizing Map Clustering Using the Anther-Specific Differentially Expressed Genes Profiles

**Figure 2:**
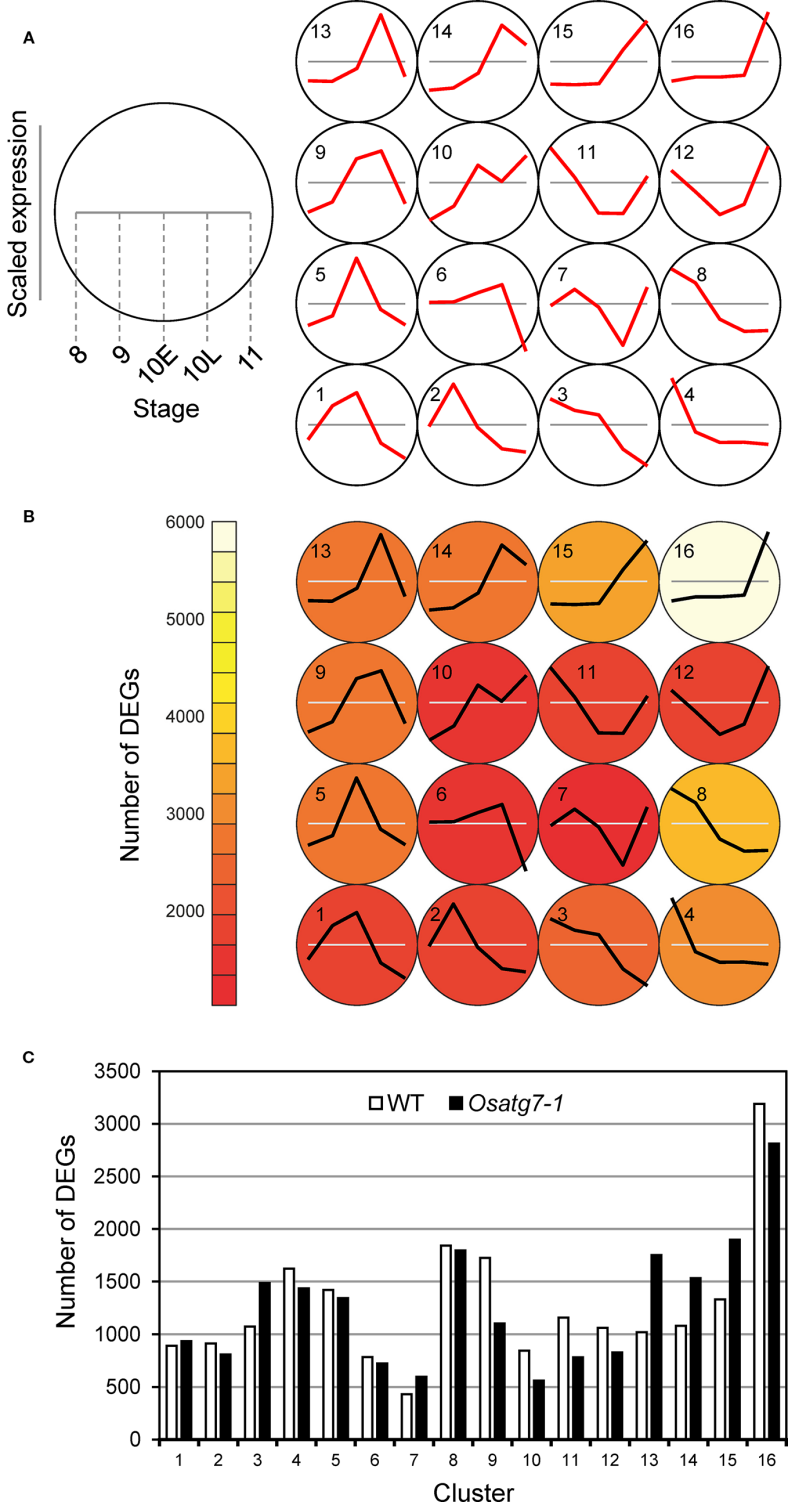
Defining ASDs depends on both anther developmental stages and autophagy disruption. Results of SOM clustering. **(A)** Line plots indicate representative expression patterns in each cluster. For SOM and diagrams, the 4 × 4 rectangular topology is shown. **(B)** Color shows number of ASDs in each cluster. **(C)** Number of ASDs in the WT and *Osatg7-1* in each cluster. The white and black bars show the numbers of ASDs in WT and *Osatg7-1* anthers, respectively.

Self-organizing map (SOM) analysis allows us to identify a subset of genes with similar expression profiles (Nakayama et al., 2018). We next performed SOM clustering to further understand the differences in expression patterns. Gene expression values from the WT and the *Osatg7-1* mutant were mean-centered and variance-scaled separately, allowing a focus on the differences in the expression patterns instead of expression magnitude. As a result, ASDs were assigned to clusters, and 16 clusters were obtained successfully (**Figures 2A, B** and **Supplementary Dataset 2**), based on box and line plots showing genes in each cluster with distinct, nonredundant expression patterns (**Supplementary Figure S3**). For instance, in cluster 9, the expression levels in ST8 and ST9 were lower than the average of the expression levels at each stage, and then the expression levels increased to ST10L. After that, their levels decreased at ST11. As shown in **Figure 2C**, the numbers of ASDs in the WT anthers were enriched in clusters 16 (3,189 DEGs; 16% of all ASDs in WT), 8 (1,843 DEGs; 9%), 9 (1,725 DEGs; 8%), and 4 (1,623 DEGs; 8%). In cluster 16, which has the most abundant of ASDs in the WT, the expression levels were lower than the average levels until ST10L, and then the expression levels drastically increased to ST11. In contrast, in the *Osatg7-1* mutant, the numbers of ASDs in cluster 9 were significantly reduced (1,104 DEGs; 5% of all ASDs in the *Osatg7-1* mutant). Conversely, the genes in clusters 15 (1,897 DEGs; 9%), 13 (1,751 DEGs; 9%), and 14 (1,533 DEGs; 8%) were increased compared with those in the WT (**Figure 2C**). The common feature of these clusters (13, 14 and 15) was that the induction of gene occurs at ST10E.

**Figure 3:**
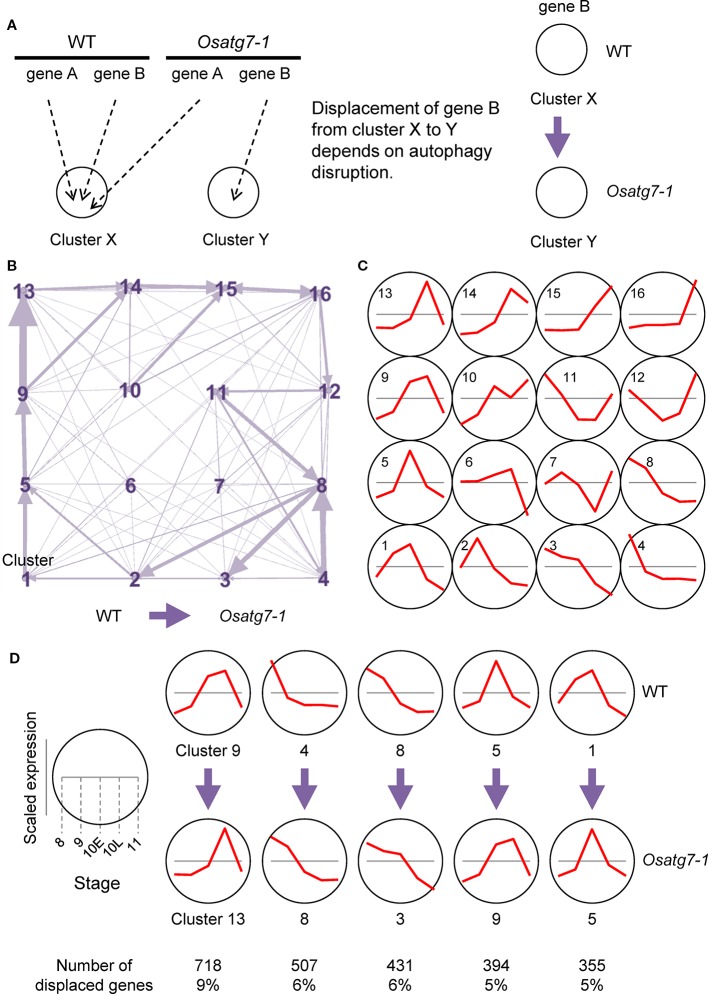
Displacement of ASDs in different clusters in the SOM clustering scheme. **(A)** A diagram demonstrating SOM clustering. The WT and *Osatg7-1* can be assigned to different clusters. **(B)** A network representation of ASDs assignment into different SOM clusters using the 7,813 displaced genes. Arrows represent displacement from the WT to *Osatg7-1*. Arrow sizes are proportional to the number of displaced ASDs. **(C)** Line plots indicate representative expression patterns in each cluster throughout developmental stages. **(D)** Major displacement directions after SOM clustering of data that were scaled separately from the WT to *Osatg7-1*. Line plots indicate representative expression patterns in each cluster.

We then focused on displaced gene sets between the WT and *Osatg7-1* mutant among clusters based on SOM clustering results from ASD data (**Figure 3A**). We extracted and visualized genes with different SOM cluster numbers in both the WT and *Osatg7-1* from SOM clustering data excluding genes that were distant from the typical cluster pattern (distance < 0.8) (**Supplementary Dataset 3**). The 7,813 displaced genes exhibited certain tendencies (**Figure 3B**; all directions from WT to *Osatg7-1*). As shown in **Figure 3D**, the numbers of displaced ASDs were enriched in clusters 4 and 9 of the WT, and these displaced gene sets were significantly enriched in displacements 9 → 13 (718 genes; 9% of displaced genes) and 4 → 8 (507 genes; 6%), suggesting that pre- and post-displacement differences in expression patterns occurs apparently at ST10E in anthers (**Figure 3C**). This trend was also confirmed by the data in **Figure 2**. Moreover, the numbers of displaced ASDs were also enriched in clusters 1, 5 and 8 of the WT, and these displaced gene sets were significantly enriched in displacements 8 → 3 (431 genes; 6%), 5 → 9 (394 genes; 5%), and 1 → 5 (355 genes; 5%) (**Figure 3D** and **Supplementary Figure S4**). These results suggest that the state of gene expression patterns of anthers is delayed in the *Osatg7-1* mutant compared with those of the WT.

**Figure 4:**
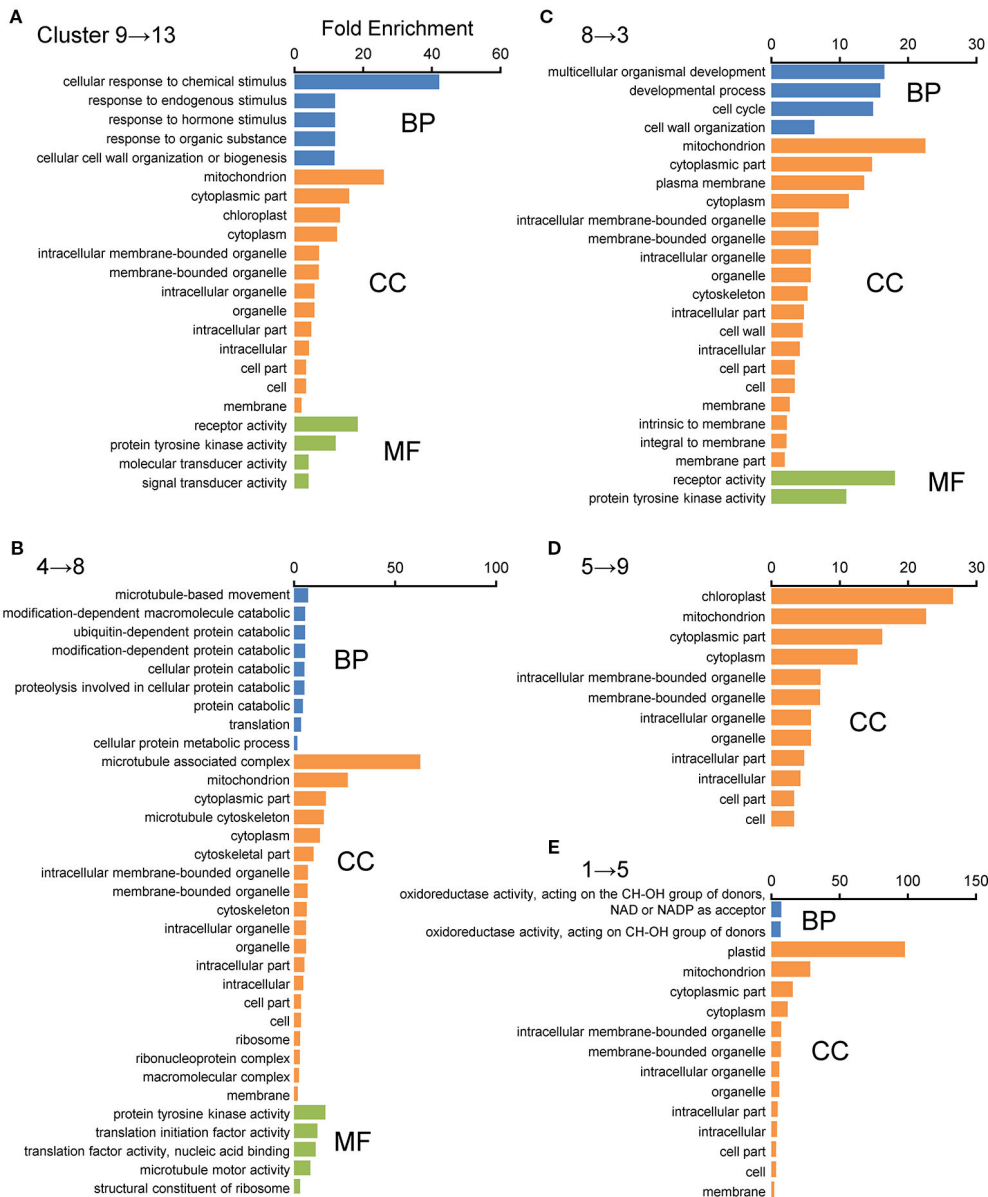
GO enrichment analyses of displacement gene sets of ASDs after SOM clustering. GO enrichment analysis was performed using the agriGO ver 2.0 program. **(A**–**E)** Top five displaced gene sets obtained from SOM clustering of ASDs (**Figure 3**) were used as datasets. Genes were categorized using the MSU7.0 gene ID (TIGR) databases as reference. The significant GO at FDR < 0.05 is shown in each cluster. Biological process (BP), molecular function (MF), and cellular component (CC).

For further characterization of these displaced gene sets between the WT and *Osatg7-1* mutant, we performed gene ontology (GO) enrichment analysis using the agriGO v2.0 program. Among these, the GO term “Mitochondrial component” as cellular components was apparently enriched in the categories of displacements 9 → 13, 4 → 8, 8 → 3, 1 → 5, and 5 → 9 (**Figure 4**). In contrast, the GO term “Plastid/Chloroplast component” was enriched in the category of displacements 1 → 5, 5 → 9, and 9 → 13. “Microtubule-associated complex” was also enriched in the category of displacements 4 → 8 (**Figure 4**). In the biological process group, the GO terms “Response to chemical stimulus” and “Secondary metabolism” were enriched in the category of displacement 9 → 13. “Cellular protein catabolic process containing ubiquitin-dependent protein” and “Cellular protein metabolic process” were enriched in the category of the displacement 4 → 8. “Cell cycle process” was enriched in the category of the displacement 8 → 3. “Cell wall organization” was enriched in the category of the displacements 9 → 13 and 8 → 3 (**Figure 4**). Moreover, in the molecular function, the GO term “Oxidoreductase activity” was ranked as the top category in displacement 1 → 5 (**Figure 4**).

Overall, the present results indicate that the state of gene expression patterns in anthers is delayed in the autophagy-deficient mutant *Osatg7-1*, and autophagy may affect mitochondrial and plastidial metabolisms during progression of tapetal PCD.

### Defining Differentially Expressed Genes Depending on Autophagy Activation in Anthers During Pollen Development

**Figure 5:**
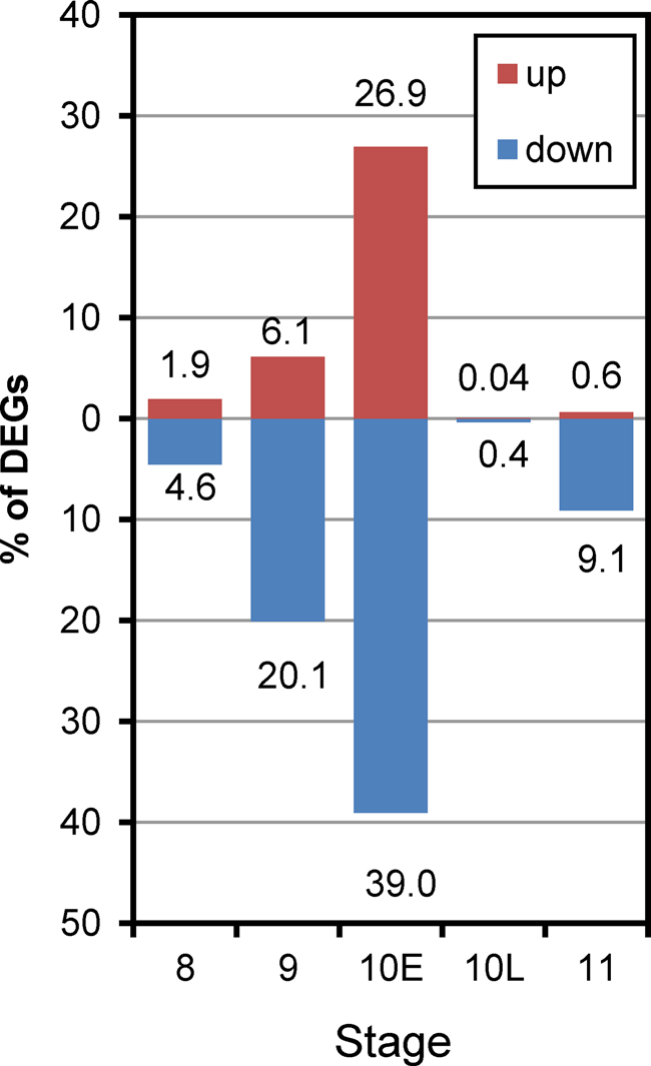
Histogram showing the number of up-regulated and down-regulated ADDs during anther developmental stages of *Osatg7-1* compared with WT. The number of genes two-fold up-regulated or down-regulated at FDR < 0.05 are plotted on the red or blue bars in the graph, respectively.

To identify DEGs affected by the activation of autophagy during pollen development, we used the R packages EdgeR. As a result, 2,359 DEGs throughout the developmental stages examined were defined as autophagy-dependent DEGs (ADDs) (FDR < 0.05) (**Supplementary Dataset 4**). In ST8 anthers, only 45 genes (1.9% in ADDs) were up-regulated and 108 (4.6%) were down-regulated in the *Osatg7-1* mutant compared with those in the WT. In ST9 anthers, 144 genes (6.1%) were up-regulated and 473 (20.1%) were down-regulated. In ST10E anthers, the effect of autophagy disruption showed the largest effect and 635 genes (26.9%) were up-regulated, while 921 (39.0%) were down-regulated. Then in ST10L anthers, only 1 gene (0.04%) was up-regulated and 9 (0.4%) were down-regulated, and in ST11 anther, 14 (0.6%) up-regulated and 215 (9.1%) down-regulated genes were identified (**Figure 5**). These results indicate that the effect of autophagy disruption on gene expression throughout pollen development was largest at ST10E, exactly at the same stage when autophagy is induced in the tapetum (Kurusu et al., 2014; Hanamata et al., 2019). Previous TEM analysis has shown that in *Osatg7-1* mutant, morphology of tapetum at ST8–9 is normal, while the tapetal collapse is significantly delayed and remaining intracellular components including mitochondria and plastid are observed at ST11 (Kurusu et al., 2014). This is consistent with the present results that disruption of autophagy affects gene expression very little at ST8, while most severely at ST10E during pollen maturation (**Figure 5**), suggesting that autophagy is crucial for timely progression of tapetal PCD, not for its initiation.

### Differences in the Transcriptome Profile between the Wild-Type and *Osatg7-1* Mutant Anthers During Pollen Maturation

**Figure 6:**
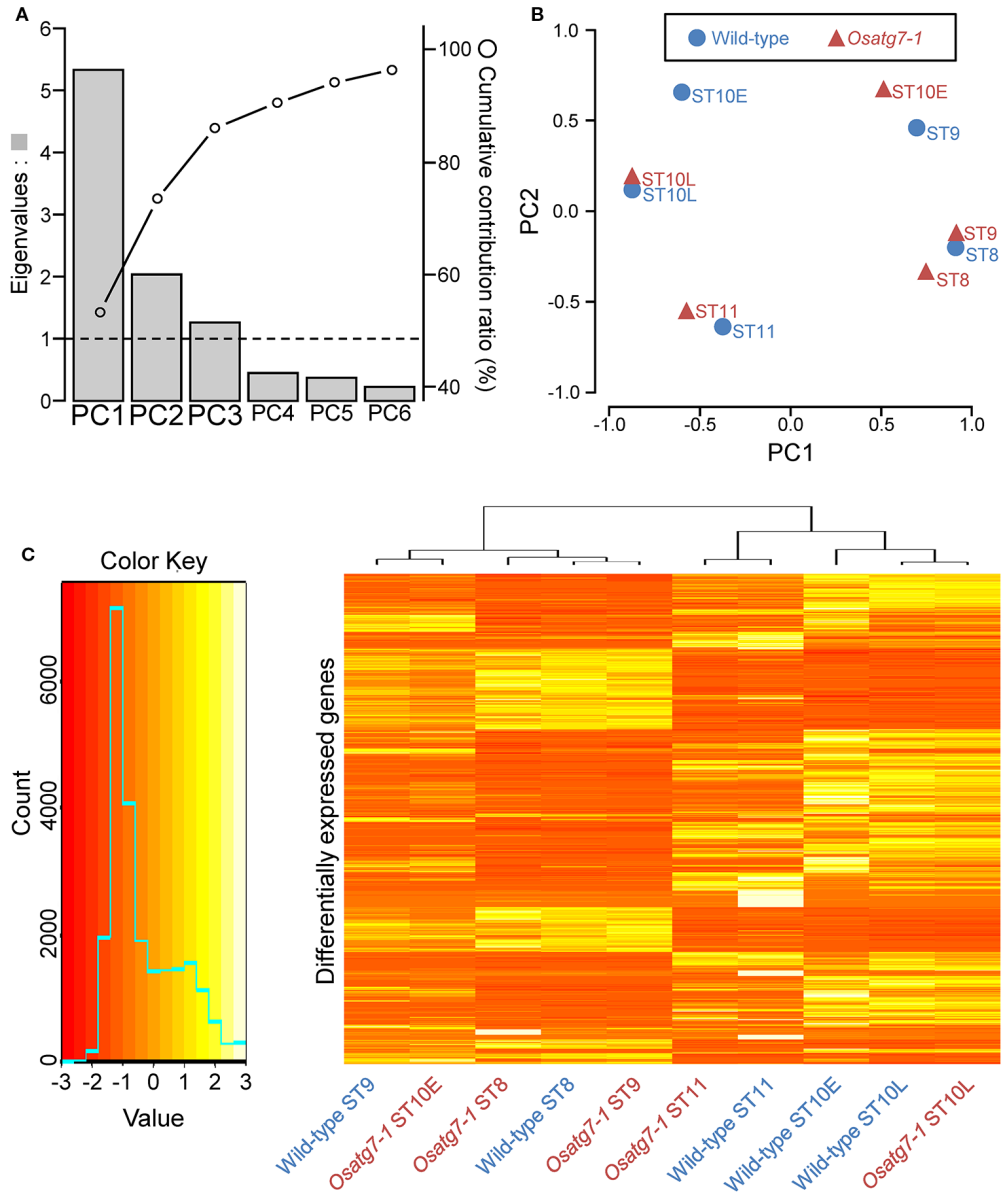
PCA of gene expression levels. **(A)** Eigenvalues and cumulative contribution ratio (%) in PCA. Bars and open circles represent eigenvalues and cumulative contribution ratio, respectively. **(B)** The global expression profile of each transcript is represented as PC1 and PC2. Note the distinct dissimilarities between the WT and *Osatg7-1* mutant at ST9 and ST10E. **(C)** Expression profiles of ADDs between the WT and *Osatg7-1* mutant during anther development. The heatmap shows log2-fold change in expression of all ADDs (2,359 genes) during anther developmental stages of *Osatg7-1* anthers compared with WT anthers.

To compare the expression profiles throughout pollen developmental stages between the WT and *Osatg7-1* mutant anthers, we performed principal component analysis (PCA) using autophagy-dependent DEGs (ADDs; 2,359 genes). Major sources of variance in the transcriptome were investigated with a PCA that considered ADDs between the WT and *Osatg7-1*. As shown in **Figure 6A**, the first component (PC1) explained 53.3% of the variation and discriminated clearly between the WT and *Osatg7-1* at ST10E. The second component (PC2) explained 20.3% of the variation and discriminated between the WT and *Osatg7-1* at ST9 (**Figures 6A, B**; **Supplementary Figure S5**). These results of PCA clearly showed that the gene expression patterns were quite similar between the WT and *Osatg7-1* at ST8, while quite different at ST9 and ST10E. Then the different patterns between the WT and *Osatg7-1* became smaller thereafter. Of note, the expression patterns in the WT at ST8 were quite similar to those of *Osatg7-1* at ST9, and the patterns of WT at ST9 were again similar to those of *Osatg7-1* at ST10E.

Using heat maps, we illustrated the significantly up- or down-regulated transcripts (*Osatg7-1 vs*. WT; 2,359 genes in **Supplementary Dataset 4**). Severe changes in the mRNA profiles throughout the reproductive period were evident, with most transcripts being consistently affected (**Figure 6C**).

### Effects of Autophagy Disruption on the Metabolism of Carbohydrates, Lipids and Phytohormones as well as Quality Control of Organelles During Pollen Maturation

**Figure 7:**
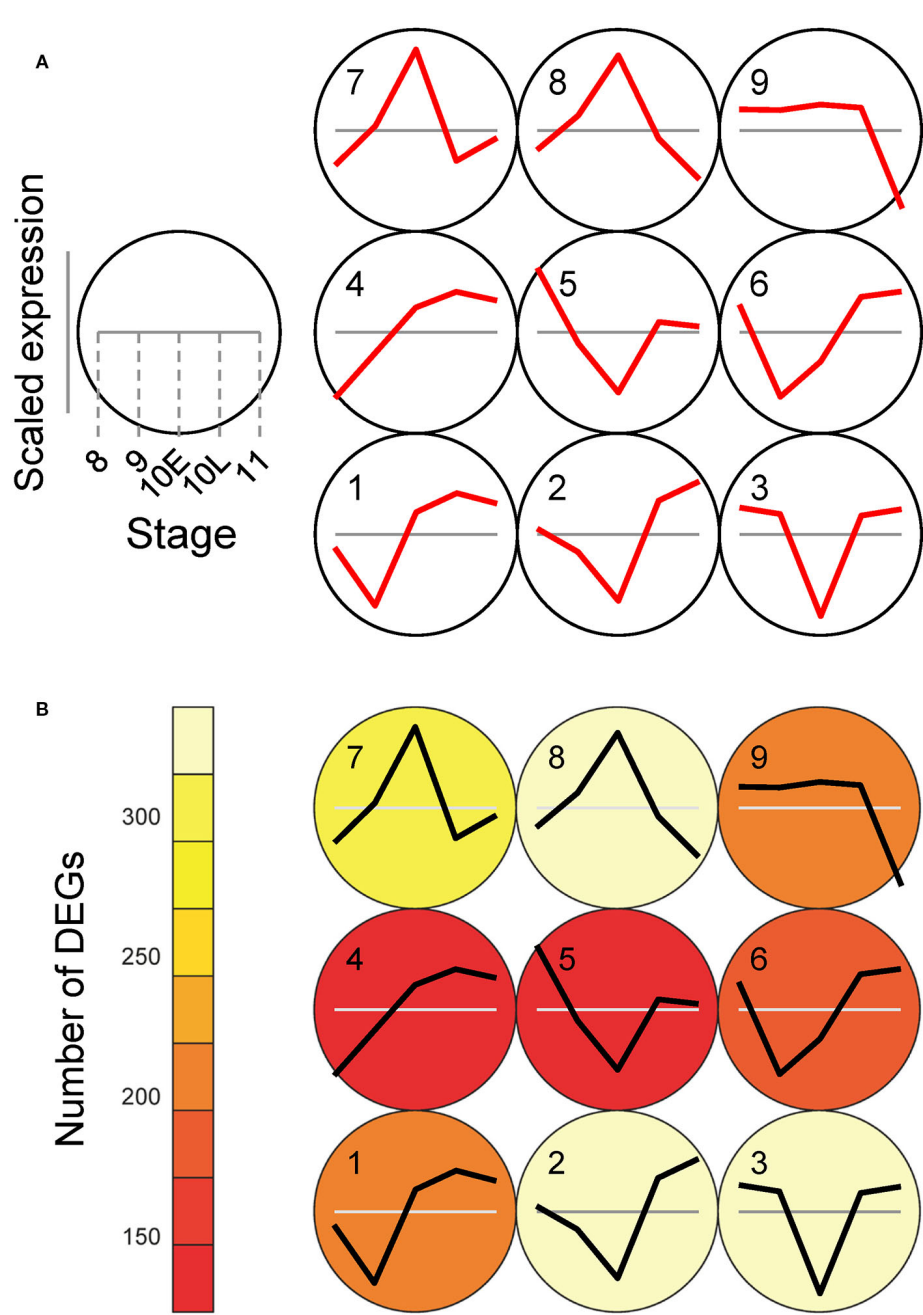
SOM clustering of gene expression levels in ADDs depend on autophagy and their expression profiles. **(A)** Results of SOM clustering using ADDs (2,149 genes with sum of cpm through all stages in the WT and *Osatg7-1* > 10). Line plots indicate representative expression patterns of *Osatg7-1* compared with WT at ST8, ST9, ST10E, ST10L and ST11 in each cluster. For SOM and diagrams, a 3 × 3 rectangular topology is shown. The WT value at each stage is shown as gray line. **(B)** The number of genes assigned to each SOM cluster. Red and white indicate low and high counts, respectively.

To identify genes under the control of autophagy during anther development precisely, we next performed SOM clustering to further understand the difference in expression patterns depending on autophagy disruption. We constructed a SOM to extract genes depending on the count data of ADDs. Gene expression values from *Osatg7-1* were mean-centered and variance-scaled using the expression data from the WT (Sum of cpm through all stages in WT and *Osatg7-1* > 10; 2,149 genes; **Supplementary Dataset 5**). As a result, all ADDs were assigned to clusters, and 9 clusters were successfully obtained (**Figures 7A, B**; **Supplementary Figure S6**). The numbers of ADDs in the *Osatg7-1* mutant were enriched in clusters 2, 3, 7, and 8, which showed peak in gene expression fluctuation is ST10E (**Figures 7A, B**; **Supplementary Figure S6**), indicating that autophagy disruption affects gene expression levels throughout anther development and most severely at ST10E.

**Figure 8:**
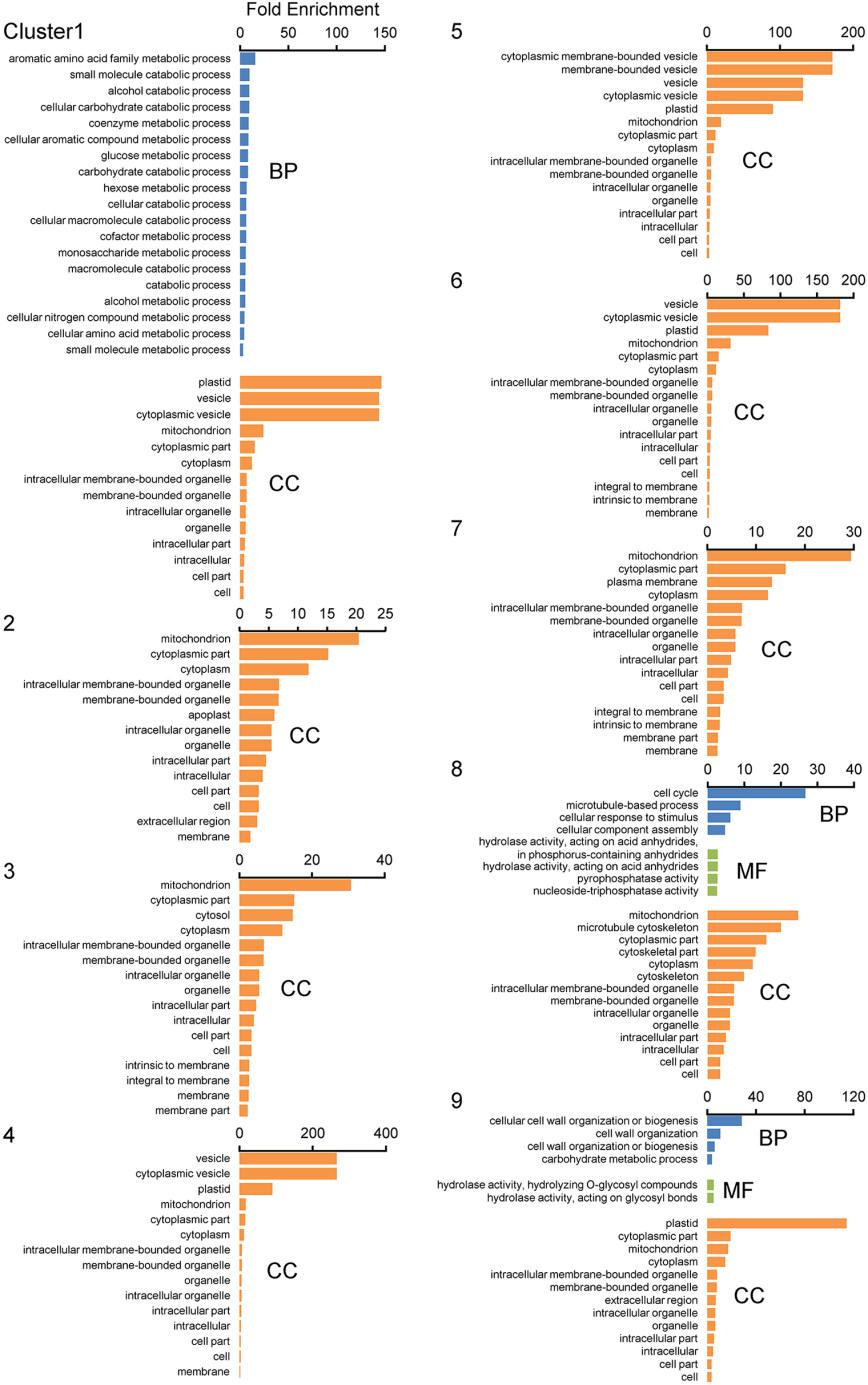
GO enrichment analyses of displacement gene sets of ADDs after SOM clustering. GO enrichment analyses were performed using the agriGO ver 2.0 program. Gene sets obtained from SOM clustering of ADDs (2,149 genes; **Figure 7**) were used as datasets. Genes were categorized using the MSU7.0 gene ID (TIGR) databases as reference. The significant GOs at FDR < 0.05 are shown in each cluster. Biological process (BP), molecular function (MF), and cellular component (CC).

For further characterization of each cluster, we performed a GO enrichment analysis with the 9 clustered gene sets (**Figure 8**). In the biological process groups, GO terms “Aromatic amino acid family metabolic process”, “Carbohydrate catabolic process”, “Cellular amino acid metabolic process”, and “Catabolic process containing carbohydrate” in cluster 1 (*q* < 0.05), GO term “Cell cycle process” in cluster 8, and “Cell wall organization” and “Carbohydrate metabolic process” in cluster 9 were enriched (**Figure 8**). Of note, GO terms “Mitochondrial component” and “Plastid/Chloroplast component” as cellular components were also enriched in almost all clusters (**Figure 8**).

**Figure 9:**
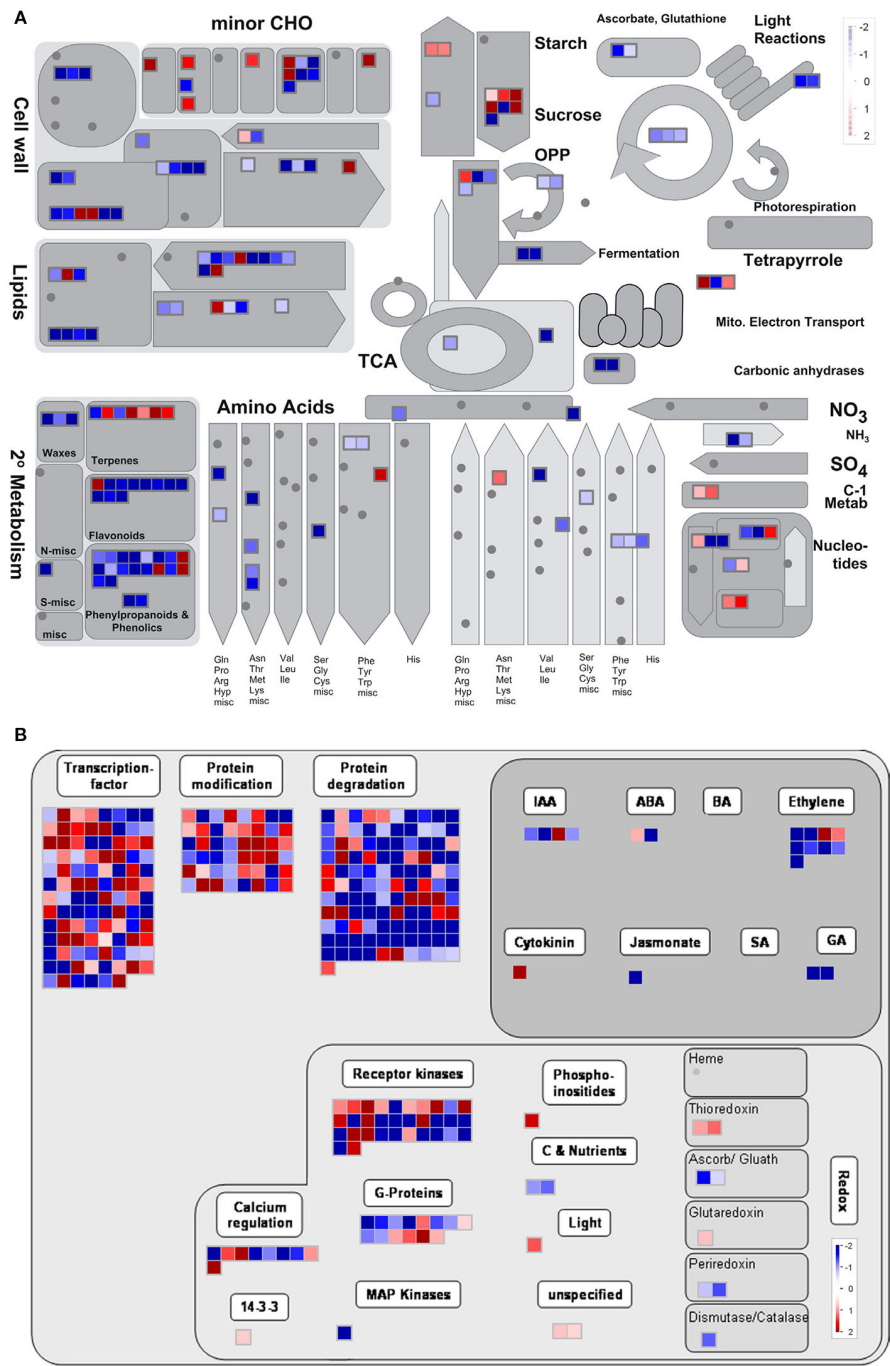
Overview of the metabolism of gene sets of ADDs by MapMan. Metabolism **(A)** and regulation **(B)** overview diagrams associated with ADDs at stage 10E. The color key represents normalized log2 values. Red represents up-regulation and blue represents down- regulation between the WT and *Osatg7-1* mutant.

We then analyzed the overview in the MapMan tool to investigate the functions of genes differentially expressed between the WT and *Osatg7-1* mutant during tapetal PCD process, especially in ST10E. Of the 1,556 gene that significantly changed (FDR < 0.05) in ST10E of ADDs, 1,500 genes were annotated and subjected to MapMan pathway analysis. As a result, 166 genes were mapped to the metabolism overview, and 351 genes were mapped to the regulation overview, respectively (**Figure 9**). Almost all metabolism-related genes are categorized as 24 genes for cell wall metabolism, 24 for lipid metabolism, 40 for secondary metabolism, 21 for amino acid metabolism, 23 for carbohydrate metabolism, 10 for nucleotide metabolism and 5 for photosystems including light reaction and Calvin-Benson cycle (**Figure 9A**).

Regulation-related genes were categorized as 102 genes for TFs, 48 genes for protein modification, 111 for protein degradation, 18 for hormone metabolism, and 57 signaling-related genes (**Figure 9B**). Hormone metabolism was further classified as 4 genes associated with IAA, 2 with abscisic acid (ABA), 9 with ethylene, 1 with cytokinin, 1 with jasmonate (JA), and 2 with gibberellin (GA) (**Figure 9B**). These results suggest that many genes affected by autophagy were associated with cell wall, lipid and carbohydrate metabolic processes as well as some phytohormones.

Hormone profiling analyses at the flowering stage have shown that endogenous levels of active-forms of GAs, were significantly lower in the *Osatg7-1*, than in the WT (Kurusu et al., 2017). Treatment with GA_4_ partially rescued the phenotype of pollen maturation in *Osatg7-1* (Kurusu et al., 2017). In addition, the expression levels of *GA20-oxidases*, which have been reported to be required for GA biosynthesis in rice (Sasaki et al., 2002), were clearly decreased in *Osatg7-1* at ST10E in comparison with that of WT (**Figure 9B** and **Supplementary Figure S7**), indicating that autophagy affects GA biosynthesis during pollen maturation in rice. Precursors of bioactive GAs are synthesized in plastids (Lange, 1998; Hedden and Phillips, 2000). Indeed, the GO term “Plastid/Chloroplast component” was enriched in almost all clusters, and photosynthesis-related genes including those involved in light reaction and Calvin-Benson cycle were decreased in the *Osatg7-1* mutant at ST10E (**Figures 8** and **9A**). Since autophagy plays an important role in the quality control of plastids including elimination and turnover of photodamaged chloroplasts (Izumi et al., 2015; Izumi et al., 2017), the lower activity of GA biosynthesis in anther may be attributed to the accumulation of abnormal plastids in *Osatg7-1* due to the disruption of autophagy. The relationship between autophagy and quality control of organelles throughout the progression of tapetal PCD should be an important future research topic.

Transcriptome analyses in the developmental process of rice anthers have shown that the genes involved in lipid and secondary metabolisms are expressed in tapetal cells and microspores at the uninucleate stages (ST9–10) (Hobo et al., 2008; Deveshwar et al., 2011). Autophagy has been shown to contribute to amino acid metabolism, lipid turnover, and maintenance of secondary metabolic pathways in nutrient-starved conditions in *Arabidopsis* and *maize* leaves (Masclaux-Daubresse et al., 2014; McLoughlin et al., 2018). In the present transcriptome analyses followed by the SOM and GO analyses of ADDs, several genes associated with both amino acid and carbohydrate metabolic/catabolic processes were enriched in clusters 1 and 9 (**Figure 8**). Moreover, in the SOM and GO analyses of ASDs, the expression patterns of these genes were enriched in cluster 4 in the WT anthers (**Figure 4**), while moved to cluster 8 in the *Osatg7-1* anthers (**Figure 4**). TEM analysis has shown that accumulation of both starch granules and lipid bodies in pollen grains are impaired in *Osatg7-1* at the mature stage (Kurusu et al., 2014). Defects in pollen maturation in *Osatg7-1* anthers may be associated with the down/delayed-regulation of carbohydrate metabolism-related genes.

**Figure 10:**
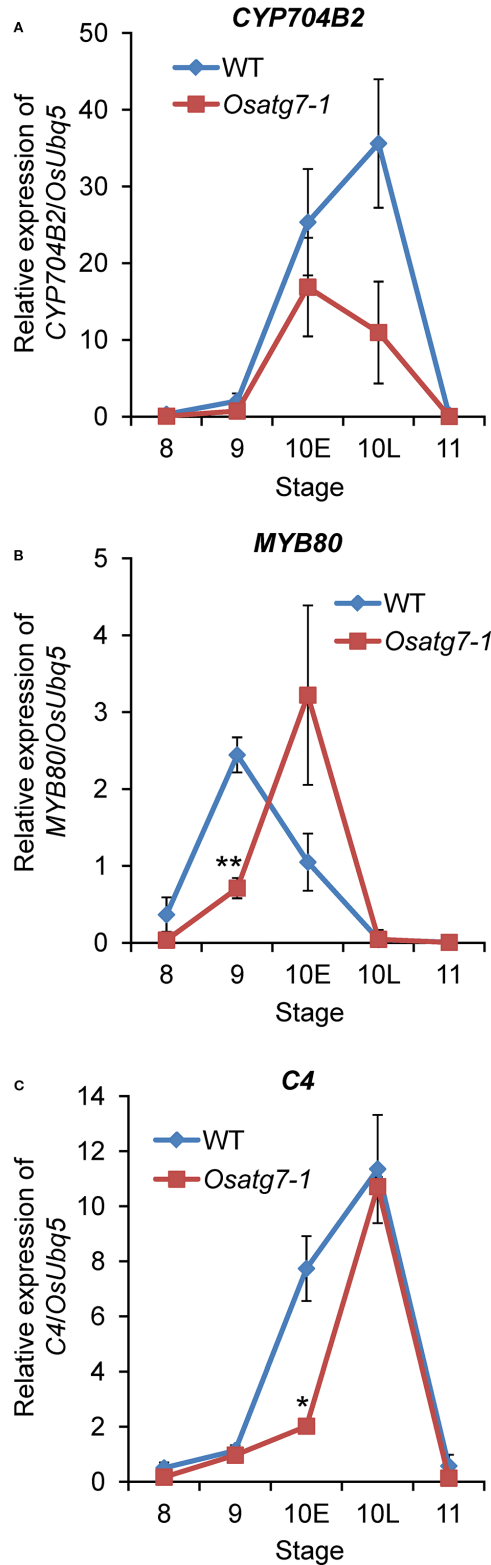
Verification of expression profiles of selected genes related to lipid metabolism by qPCR analysis. Quantitative expression levels of *CYP704B2* **(A)** and *MYB80* **(B)** and *C4* **(C)** during anther developmental stages. The amount of each mRNA was calculated from the threshold point located in the log-linear range of RT-PCR. The relative level of each gene in the WT anthers at tetrad stage (ST8) was standardized as 1. Data are the means ± SE of three independent experiments. *P < 0.05, **P < 0.01; significantly different from the controls.

**Figure 11:**
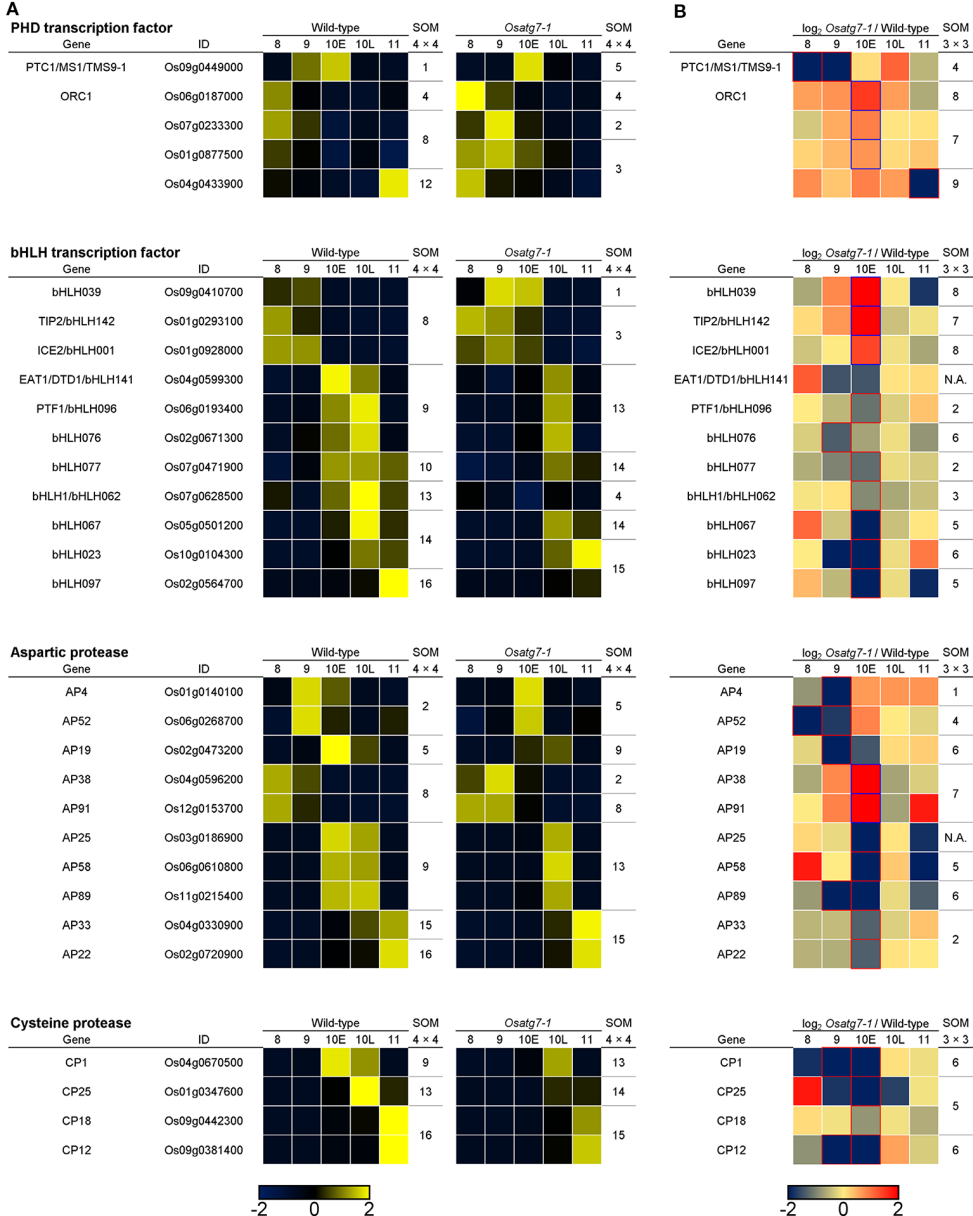
Expression profiles and SOM clustering of ADDs involved in tapetal PCD between the WT and *Osatg7-1* mutant anthers during pollen development. **(A)** The heatmap and SOM clustering show in scaled expression patterns of tapetal PCD-related genes of rice during anther developmental stages in both WT and *Osatg7-1* mutant. **(B)** The heatmap shows log2-fold change in expression of ADDs involved in tapetal PCD during anther developmental stages of *Osatg7-1* compared with WT. For SOM clustering of ADDs, both 4 × 4 **(A)** and 3 × 3 **(B)** rectangular topologies are used, and extracted genes are assigned to each SOM cluster, respectively.

Lipidic exine synthesis is an important component of the pollen wall in rice and *Arabidopsis* (Yang et al., 2007). At the flowering stage, development of the coat structure of pollen grains and accumulation of lipid bodies in pollen grains are impaired in *Osatg7-1* (Kurusu et al., 2014). Moreover, MapMan analysis clearly showed that autophagy disruption affects the expression of genes involved in lipid and cell wall metabolic processes at ST10E in anthers (**Figure 9**). Therefore, the expression patterns of several marker genes related to pollen sporopollenin biosynthesis were checked by RNA-seq as well as qPCR analysis (**Figure 10**). Induced expression of *CYP704B2,* which has been reported to be required for sporopollenin and pollen wall biosynthesis in rice (Li et al., 2010), was lower at ST10E–10L in the *Osatg7-1* than WT (**Figure 10**). Moreover, the expression of a rice homolog of MYB80, which is required for tapetal PCD and anther development in *Arabidopsis* (Phan et al., 2011), was also lower and delayed at ST9–10E (**Figure 10**). Induced expression of *PTC1* encoding a key TF regulator of sporopollenin biosynthesis, was also delayed in the *Osatg7-1* at ST9 (**Figure 11**), suggesting the involvement of autophagy in sporopollenin biosynthesis and pollen wall formation during pollen maturation. Moreover, a lipid transfer protein of rice, anther specific protein 4 (C4), is specifically expressed in tapetal cells (Tsuchiya et al., 1994) and is able to transport lipid molecules such as fatty acids from tapetal cells to developing microspores. The induction of *C4* gene was also suppressed in the *Osatg7-1* compared with WT at ST10E (**Figure 10**). These results suggest that the immature pollen phenotype of *Osatg7-1* is attributed to the defects in timely transport of lipids from the tapetum cells to developing microspores, which may lead to less accumulation of lipid contents in the formation of shrunken pollen in the mutant.

### Effects of Autophagy Disruption on the Progression of Tapetal PCD During Pollen Maturation

Appropriate temporal regulation of tapetal PCD is vital for normal pollen development in plants. The signal initiating tapetal PCD has been suggested to be first produced during the tetrad stage (ST8) (Kawanabe et al., 2006). A transcriptional regulatory network as well as activation of proteases is also known to play a key role in the progression of tapetal PCD, and some key genes involved in the tapetal PCD include the PHD-finger transcription factors (PHD-TFs) and the bHLH transcription factors (bHLH-TFs) as well as aspartic proteases (APs) and cysteine proteases (CPs) (Niu et al., 2013; Ono et al., 2018; Yang et al., 2019).

**Figure 12:**
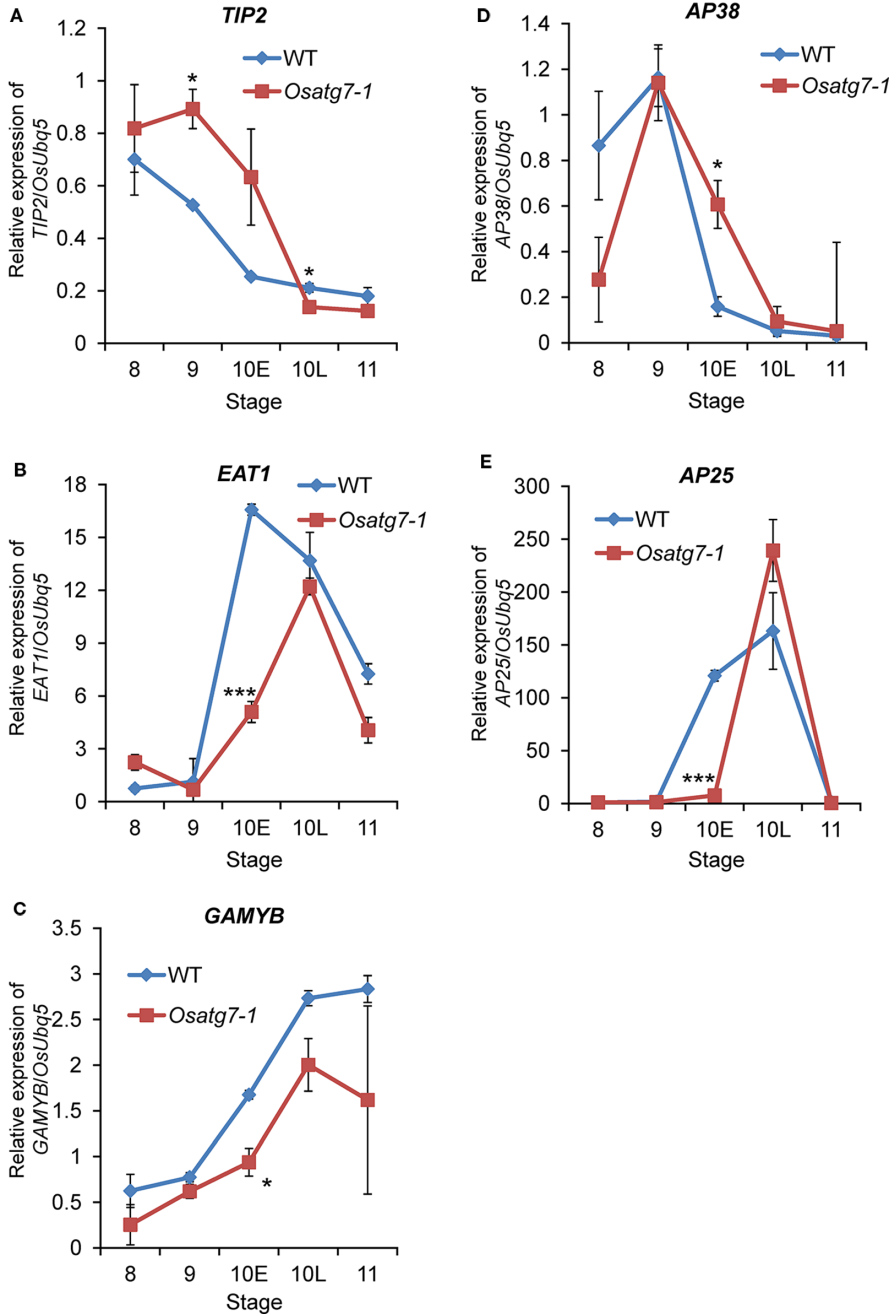
Verification of expression profiles of selected genes related to tapetal PCD progression and pollen maturation by qPCR analysis. Quantitative expression levels of *TIP2* **(A)** and *EAT1* **(B)** and *GAMYB* **(C)** and *AP38* **(D)** and *AP25* **(E)** during anther developmental stages. The amount of each mRNA was calculated from the threshold point located in the log-linear range of RT-PCR. The relative level of each gene in the WT anthers at tetrad stage (ST8) was standardized as 1. Data are the means ± SE of three independent experiments. *P < 0.05, ***P < 0.001; significantly different from the controls.

To investigate the effects of autophagy disruption on the transcriptional regulatory networks during tapetal PCD process, we searched the expression profiles of *PHD-TFs* and *bHLH-TFs* as well as *CPs* and *APs* genes in rice (Chen et al., 2009; Wang et al., 2018). We extracted 5 *PHD-TFs* and 10 *bHLH-TFs* as well as 9 *APs* and 4 *CPs* (ADDs; FDR < 0.05). The expression level of *PTC1*, which has been reported to be required for rice tapetal PCD (Li et al., 2011), was decreased and delayed from ST8 to ST9 in the *Osatg7-1* compared with WT (**Figure 11**). The induction of selected *bHLH-TFs* was also decreased in the *Osatg7-1* anthers throughout tapetal PCD (**Figure 11**), and these characteristic expression patterns of some key genes, such as *EAT1* involved in PCD progression were also confirmed by qPCR analysis (**Figure 12**). Moreover, the induction of *GAMYB*, which has been reported to be required for tapetal PCD and pollen development in rice (Aya et al., 2009), was also suppressed at the ST10–11 in the anthers of *Osatg7-1*, suggesting that autophagy disruption alters the expression patterns of the key regulatory TFs associated with PCD throughout the progression of tapetal PCD.

EAT1 is known to trigger tapetal PCD by regulating the expression of two aspartic proteases (AP25 and AP37) at the young microspore stage (Niu et al., 2013). The induction of *EAT1* gene was suppressed at the ST10E in the anthers of *Osatg7-1*. Previous TEM analysis indicated the delay of tapetal PCD in *Osatg7-1* anthers (Kurusu et al., 2014), which was correlated with the down-regulation of *EAT1* expression at pollen developmental stages (**Figures 11** and **12**). Moreover, the expression levels of 4 *CP* genes were significantly down-regulated in *Osatg7-1* anthers compared with those of the WT throughout tapetal PCD progression (**Figure 11**). The induction of *AP25*, which has been reported to be required for rice tapetal PCD (Niu et al., 2013), was also delayed and decreased at ST9-10E in *Osatg7-1* compared with the WT (**Figures 11** and **12**). Wild-type anthers exhibited strong induction of *EAT1*, *AP25* as well as *AP38* at ST9 to turn on timely progression of tapetal PCD (**Figures 11** and **12**). As shown in **Figure 7**, the expression patterns of almost all ADDs apparently fluctuated at ST10E. These trends were also confirmed using heatmap analysis, SOM clustering as well as qPCR analysis (**Figure 12**). Thus, the impairment and delay of tapetal PCD in the autophagy-deficient mutant may at least in part be attributed to the reduced expression of these genes involved in the progression of tapetal PCD at ST9-10 in anthers.

### Future Perspectives

Autophagy-deficient mutants in *Arabidopsis* and rice are reported to be hypersensitive to oxidative stress (Xiong et al., 2007a; Xiong et al., 2007b; Shin et al., 2010), suggesting that autophagy plays an important role in oxidative stress responses during anther development. As an important protein quality control mechanism, autophagy has been suggested to play a critical role in the degradation of these misfolded/denatured and potentially highly toxic proteins or protein aggregates (Kraft et al., 2010; Johansen and Lamark, 2011; Shaid et al., 2013). Since autophagy is involved in the turnover of damaged mitochondria and chloroplasts (Izumi et al., 2015; Izumi et al., 2017), autophagy disruption in anthers could cause over-accumulation of reactive oxygen species (ROS) under various environmental stress. Autophagy induced during tapetal PCD may play critical roles in the quality control of organelles and environmental stress responses including oxidative stress during pollen maturation.

## Data Availability Statement

The datasets generated for this study can be found in the DRA008977.

## Author Contributions

TK and KK designed the research. SH, JS, SO, KO, TF, and SK performed the research. SH, JS, and SK analyzed the data. TK, SH, JS, K-IN, SK, and KK wrote the manuscript.

## Funding

This work was supported in part by Grants-in-Aid from the Ministry of Education, Culture, Sports, Science and Technology for challenging Exploratory Research (17K19274) to KK, for Scientific Research on Innovative Areas (16H01207) to KK, for Scientific Research (C) (18K05562) to TK as well as KAKENHI (16H01472, 16K07408, 18H04787, and 18H04844) to SK, for the MEXT Supported Program (S1511023) of the Strategic Research Foundation at Private Universities from the Ministry of Education to SK, by the Sumitomo Foundation (181245) to TK, and by the NIG-JOINT (39A2019) to KK.

## Conflict of Interest

The authors declare that the research was conducted in the absence of any commercial or financial relationships that could be construed as a potential conflict of interest.

## References

[B1] Anders S. Pyl P. T. Huber W. (2015). HTSeq-a Python framework to work with high-throughput sequencing data. Bioinformatics 31, 166–169. 10.1093/bioinformatics/btu638 25260700 PMC4287950

[B2] Ariizumi T. Toriyama K. (2011). Genetic regulation of sporopollenin synthesis and pollen exine development. Annu. Rev. Plant Biol. 62, 437–460. 10.1146/annurev-arplant-042809-112312 21275644

[B3] Avin-Wittenberg T. Honig A. Galili G. (2012). Variations on a theme: plant autophagy in comparison to yeast and mammals. Protoplasma 249, 285–299. 10.1007/s00709-011-0296-z 21660427

[B4] Aya K. Ueguchi T. M. Kondo M. Hamada K. Yano K. Nishimura M. (2009). Gibberellin modulates anther development in rice *via the* transcriptional regulation of *GAMYB* . Plant Cell 21, 1453–1472. 10.1105/tpc.108.062935 19454733 PMC2700530

[B5] Bastian M. Heymann S. Jacomy M. (2009). “An open source software for exploring and manipulating networks,” in Proceedings of the Third International Conference on Weblogs and Social Media (Menlo Park, CA: AAAI Press), 361–362.

[B6] Bokvaj P. Hafidh S. Honys D. (2015). Transcriptome profiling of male gametophyte development in *Nicotiana tabacum* . Genomic Data 3, 106–111. 10.1016/j.gdata.2014.12.002 PMC4535457 26484158

[B7] Chen J. Ouyang Y. Wang L. Xie W. Zhang Q. (2009). Aspartic proteases gene family in rice: gene structure and expression, predicted protein features and phylogenetic relation. Gene 442, 108–118. 10.1016/j.gene.2009.04.021 19409457

[B8] Chitwood D. H. Maloof J. N. Sinha N. R. (2013). Dynamic transcriptomic profiles between tomato and a wild relative reflect distinct developmental architectures. Plant Physiol. 162, 537–552. 10.1104/pp.112.213546 23585653 PMC3668051

[B9] Chung T. Suttangkakul A. Vierstra R. D. (2009). The ATG autophagic conjugation system in maize: ATG transcripts and abundance of the ATG8-lipid adduct are regulated by development and nutrient availability. Plant Physiol. 149, 220–234. 10.1104/pp.108.126714 18790996 PMC2613746

[B10] Deveshwar P. Bovill W. D. Sharma R. Able J. A. Kapoor S. (2011). Analysis of anther transcriptomes to identify genes contributing to meiosis and male gametophyte development in rice. BMC Plant Biol. 11, e78. 10.1186/1471-2229-11-78 PMC3112077 21554676

[B11] Feng B. M. Lu D. H. Ma X. Peng Y. B. Sun Y. J. Ning G. (2012). Regulation of the *Arabidopsis* anther transcriptome by DYT1 for pollen development. Plant J. 72, 612–624. 10.1111/j.1365-313X.2012.05104.x 22775442

[B12] Fu Z. Yu J. Cheng X. Zong X. Xu J. Chen M. (2014). The rice basic helix-loop-helix transcription factor TDR INTERACTING PROTEIN2 is a central switch in early anther development. Plant Cell 26, 1512–1524. 10.1105/tpc.114.123745 24755456 PMC4036568

[B13] Ghiglione H. O. Gonzalez F. G. Serrago R. Maldonado S. B. Chilcott C. Cura J. A. (2008). Autophagy regulated by day length determines the number of fertile florets in wheat. Plant J. 55, 1010–1024. 10.1111/j.1365-313X.2008.03570.x 18547393

[B14] Gu J. N. Zhu J. Yu Y. Teng X. D. Lou Y. Xu X. F. (2014). DYT1 directly regulates the expression of *TDF1* for tapetum development and pollen wall formation in *Arabidopsis* . Plant J. 80, 1005–1013. 10.1111/tpj.12694 25284309

[B15] Hanamata S. Kurusu T. Kuchitsu K. (2014). Roles of autophagy in reproductive development in plants. Front. Plant Sci. 5, e457. 10.3389/fpls.2014.00457 PMC4163999 25309556

[B16] Hanamata S. Sawada J. Toh B. Ono S. Ogawa K. Fukunaga T. (2019). Monitoring autophagy in rice tapetal cells during pollen maturation. Plant Biotechnol. (Tokyo) 36, 99–105. 10.5511/plantbiotechnology.19.0417a 31768110 PMC6847784

[B17] Hedden P. Phillips A. L. (2000). Gibberellin metabolism: new insights revealed by the genes. Trends Plant Sci. 5, 523–530. 10.1016/S1360-1385(00)01790-8 11120474

[B18] Higginson T. Li S. F. Parish R. W. (2003). AtMYB103 regulates tapetum and trichome development in *Arabidopsis thaliana* . Plant J. 35, 177–192. 10.1046/j.1365-313X.2003.01791.x 12848824

[B19] Hobo T. Suwabe K. Aya K. Suzuki G. Yano K. Ishimizu T. (2008). Various spatiotemporal expression profiles of anther-expressed genes in rice. Plant Cell Physiol. 49, 1417–1428. 10.1093/pcp/pcn128 18776202 PMC2566926

[B20] Honys D. Twell D. (2004). Transcriptome analysis of haploid male gametophyte development in *Arabidopsis* . Genome Biol. 5, R85. 10.1186/gb-2004-5-11-r85 15535861 PMC545776

[B21] Huang M. D. Hsing Y. Huang A. H. (2011). Transcriptomes of the anther sporophyte: availability and uses. Plant Cell Physiol. 52, 1459–1466. 10.1093/pcp/pcr088 21743085 PMC3172567

[B22] Ishida H. Yoshimoto K. Izumi M. Reisen D. Yano Y. Makino A. (2008). Mobilization of rubisco and stroma-localized fluorescent proteins of chloroplasts to the vacuole by an *ATG* gene-dependent autophagic process. Plant Physiol. 148, 142–155. 10.1104/pp.108.122770 18614709 PMC2528122

[B23] Izumi M. Hidema J. Wada S. Kondo E. Kurusu T. Kuchitsu K. (2015). Establishment of monitoring methods for autophagy in rice reveals autophagic recycling of chloroplasts and root plastids during energy limitation. Plant Physiol. 167, 1307–1320. 10.1104/pp.114.254078 25717038 PMC4378162

[B24] Izumi M. Ishida H. Nakamura S. Hidema J. (2017). Entire photodamaged chloroplasts are transported to the central vacuole by autophagy. Plant Cell 29, 377–394. 10.1105/tpc.16.00637 28123106 PMC5354188

[B25] Johansen T. Lamark T. (2011). Selective autophagy mediated by autophagic adapter proteins. Autophagy 7, 279–296. 10.4161/auto.7.3.14487 21189453 PMC3060413

[B26] Jung K. H. An G. (2012). Application of MapMan and RiceNet drives systematic analyses of the early heat stress transcriptome in rice seedlings. J. Plant Biol. 55, 436–449. 10.1007/s12374-012-0270-0

[B27] Jung K. H. Han M. J. Lee Y. S. Kim Y. W. Hwang I. Kim M. J. (2005). Rice *Undeveloped Tapetum1* is a major regulator of early tapetum development. Plant Cell 17, 2705–2722. 10.1105/tpc.105.034090 16141453 PMC1242267

[B28] Kawanabe T. Ariizumi T. Kawai-Yamada M. Uchimiya H. Toriyama K. (2006). Abolition of the tapetum suicide program ruins microsporogenesis. Plant Cell Physiol. 47, 784–787. 10.1093/pcp/pcj039 16565524

[B29] Kim Y. J. Zhang D. (2018). Molecular control of male fertility for crop hybrid breeding. Trends Plant Sci. 23, 53–65. 10.1016/j.tplants.2017.10.001 29126789

[B30] Kim D. Pertea G. Trapnell C. Pimentel H. Kelley R. Salzberg S. L. (2013). TopHat2: accurate alignment of transcriptomes in the presence of insertions, deletions and gene fusions. Genome Biol. 14, R36. 10.1186/gb-2013-14-4-r36 23618408 PMC4053844

[B31] Ko S. S. Li M. J. Ku M. S. B. Ho Y. C. Lin Y. J. Chuang M. H. (2014). The bHLH142 transcription factor coordinates with TDR1 to modulate the expression of *EAT1* and regulate pollen development in rice. Plant Cell 26, 2486–2504. 10.1105/tpc.114.126292 24894043 PMC4114947

[B32] Kohonen T. (1982). Self-organized formation of topologically correct feature Maps. Biol. Cybern. 43, 59–69. 10.1007/BF00337288

[B33] Kraft C. Peter M. Hofmann K. (2010). Selective autophagy: ubiquitin-mediated recognition and beyond. Nat. Cell Biol. 12, 836–841. 10.1038/ncb0910-836 20811356

[B34] Kurusu T. Kuchitsu K. (2017). Autophagy, programmed cell death and reactive oxygen species in sexual reproduction in plants. J. Plant Res. 130, 491–499. 10.1007/s10265-017-0934-4 28364377

[B35] Kurusu T. Koyano T. Hanamata S. Kubo T. Noguchi Y. Yagi C. (2014). OsATG7 is required for autophagy-dependent lipid metabolism in rice postmeiotic anther development. Autophagy 10, 878–888. 10.4161/auto.28279 24674921 PMC5119067

[B36] Kurusu T. Hanamata S. Kuchitsu K. (2016). Quantitative live cell imaging of autophagic flux and roles of autophagy in reproductive development in plants. Bioimages 24, 1–11. 10.11169/bioimages.24.1

[B37] Kurusu T. Koyano T. Kitahata N. Kojima M. Hanamata S. Sakakibara H. (2017). Autophagy-mediated regulation of phytohormone metabolism during rice anther development. Plant Signal. Behav. 12, e1365211. 10.1080/15592324.2017.1365211 28873038 PMC5640179

[B38] Kwon S. I. Cho H. J. Park O. K. (2010). Role of *Arabidopsis* RabG3b and autophagy in tracheary element differentiation. Autophagy 6, 1187–1189. 10.4161/auto.6.8.13429 20861670

[B39] Lange T. (1998). Molecular biology of gibberellin synthesis. Planta 204, 409–419. 10.1007/s004250050274 9684365

[B40] Li F. Vierstra R. D. (2012). Autophagy: a multifaceted intracellular system for bulk and selective recycling. Trends Plant Sci. 17, 526–537. 10.1016/j.tplants.2012.05.006 22694835

[B41] Li N. Zhang D. S. Liu H. S. Yin C. S. Li X. X. Liang W. Q. (2006). The rice tapetum degeneration retardation gene is required for tapetum degradation and anther development. Plant Cell 18, 2999–3014. 10.1105/tpc.106.044107 17138695 PMC1693939

[B42] Li H. Pinot F. Sauveplane V. Werck-Reichhart D. Diehl P. Schreiber L. (2010). Cytochrome P450 family member CYP704B2 catalyzes the ω-hydroxylation of fatty acids and is required for anther cutin biosynthesis and pollen exine formation in rice. Plant Cell 22, 173–190. 10.1105/tpc.109.070326 20086189 PMC2828706

[B43] Li H. Yuan Z. Vizcay-Barrena G. Yang C. Liang W. Zong J. (2011). *PERSISTENT TAPETAL CELL1* encodes a PHD-Finger protein that is required for tapetal cell death and pollen development in rice. Plant Physiol. 156, 615–630. 10.1104/pp.111.175760 21515697 PMC3177263

[B44] Masclaux-Daubresse C. Clément G. Anne P. Routaboul J. M. Guiboileau A. Soulay F. (2014). Stitching together the multiple dimensions of autophagy using metabolomics and transcriptomics reveals impacts on metabolism, development, and plant responses to the environment in *Arabidopsis* . Plant Cell 26, 1857–1877. 10.1105/tpc.114.124677 24808053 PMC4079355

[B45] McCarthy D. J. Chen Y. Smyth G. K. (2012). Differential expression analysis of multifactor RNA-Seq experiments with respect to biological variation. Nucleic Acids Res. 40, 4288–4297. 10.1093/nar/gks042 22287627 PMC3378882

[B46] McLoughlin F. Augustine R. C. Marshall R. S. Li F. Kirkpatrick L. D. Otegui M. S. (2018). *Maize* multi-omics reveal roles for autophagic recycling in proteome remodelling and lipid turnover. Nat. Plants 4, 1056–1070. 10.1038/s41477-018-0299-2 30478358

[B47] Mizushima N. Komatsu M. (2011). Autophagy: renovation of cells and tissues. Cell 147, 728–741. 10.1016/j.cell.2011.10.026 22078875

[B48] Murashige T. Skoog F. (1962). A revised medium for rapid growth and bioassays with tobacco tissue cultures. Physiol. Plant 15, 473–492. 10.1111/j.1399-3054.1962.tb08052.x

[B49] Nakayama H. Sakamoto T. Okegawa Y. Kaminoyama K. Fujie M. Ichihashi Y. (2018). Comparative transcriptomics with self-organizing map reveals cryptic photosynthetic differences between two accessions of North American Lake cress. Sci. Rep. 8, e3302. 10.1038/s41598-018-21646-w PMC5818620 29459626

[B50] Niu N. Liang W. Yang X. Jin W. Wilson Z. A. Hu J. (2013). EAT1 promotes tapetal cell death by regulating aspartic proteases during male reproductive development in rice. Nat. Commun. 4, e1445. 10.1038/ncomms2396 23385589

[B51] Ono S. Liu H. Tsuda K. Fukai E. Tanaka K. Sasaki T. (2018). EAT1 transcription factor, a non-cell-autonomous regulator of pollen production, activates meiotic small RNA biogenesis in rice anther tapetum. PloS Genet. 14, e1007238. 10.1371/journal.pgen.1007238 29432414 PMC5825165

[B52] Phan H. A. Iacuone S. Li S. F. Parish R. W. (2011). The MYB80 transcription factor is required for pollen development and the regulation of tapetal programmed cell death in *Arabidopsis thaliana* . Plant Cell 23, 2209–2224. 10.1105/tpc.110.082651 21673079 PMC3160043

[B53] Robinson M. D. McCarthy D. J. Smyth G. K. (2010). edgeR: a Bioconductor package for differential expression analysis of digital gene expression data. Bioinformatics 26, 139–140. 10.1093/bioinformatics/btp616 19910308 PMC2796818

[B54] Rutley N. Twell D. (2015). A decade of pollen transcriptomics. Plant Reprod. 28, 73–89. 10.1007/s00497-015-0261-7 25761645 PMC4432081

[B55] Sasaki A. Ashikari M. Ueguchi-Tanaka M. Itoh H. Nishimura A. Swapan D. (2002). Green revolution: a mutant gibberellin-synthesis gene in rice. Nature 416, 701–702. 10.1038/416701a 11961544

[B56] Sera Y. Hanamata S. Sakamoto S. Ono S. Kaneko K. Mitsui Y. (2019). Essential roles of autophagy in metabolic regulation in endosperm development during rice seed maturation. Sci. Rep. 9, 18544. 10.1038/s41598-019-54361-1 31811157 PMC6898296

[B57] Shaid S. Brandts C. H. Serve H. Dikic I. (2013). Ubiquitination and selective autophagy. Cell Death Differ. 20, 21–30. 10.1038/cdd.2012.72 22722335 PMC3524631

[B58] Shibata M. Oikawa K. Yoshimoto K. Kondo M. Mano S. Yamada K. (2013). Highly oxidized peroxisomes are selectively degraded *via* autophagy in *Arabidopsis* . Plant Cell 25, 4967–4983. 10.1105/tpc.113.116947 24368788 PMC3903999

[B59] Shin J. H. Yoshimoto K. Ohsumi Y. Jeon J. S. An G. (2010). OsATG10b, an autophagosome component, is needed for cell survival against oxidative stresses in rice. Mol. Cells 27, 67–74. 10.1007/s10059-009-0006-2 19214435

[B60] Sorensen A. M. Krober S. Unte U. S. Huijser P. Dekker K. Saedler H. (2003). The *Arabidopsis ABORTED MICROSPORES* (*AMS*) gene encodes a MYC class transcription factor. Plant J. 33, 413–423. 10.1046/j.1365-313X.2003.01644.x 12535353

[B61] Thompson A. R. Doelling J. H. Suttangkakul A. Vierstra R. D. (2005). Autophagic nutrient recycling in *Arabidopsis* directed by the ATG8 and ATG12 conjugation pathways. Plant Physiol. 138, 2097–2110. 10.1104/pp.105.060673 16040659 PMC1183398

[B62] Tsuchiya T. Toriyama K. Ejiri S. Hinata K. (1994). Molecular characterization of rice genes specifically expressed in the anther tapetum. Plant Mol. Biol. 26, 1737–1746. 10.1007/BF00019488 7858214

[B63] Vizcay-Barrena G. Wilson Z. A. (2006). Altered tapetal PCD and pollen wall development in the *Arabidopsis ms1* mutant. J. Exp.Bot. 57, 2709–2717. 10.1093/jxb/erl032 16908508

[B64] Wada S. Hayashida Y. Izumi M. Kurusu T. Hanamata S. Kanno K. (2015). Autophagy supports biomass production and nitrogen use efficiency at the vegetative stage in rice. Plant Physiol. 168, 60–73. 10.1104/pp.15.00242 25786829 PMC4424030

[B65] Wang W. Zhou X. M. Xiong H. X. Mao W. Y. Zhao P. Sun M. X. (2018). Papain-like and legumain-like proteases in rice: genome-wide identification, comprehensive gene feature characterization and expression analysis. BMC Plant Biol. 18, 87. 10.1186/s12870-018-1298-1 29764367 PMC5952849

[B66] Wehrens R. Buydens L. M. C. (2007). Self- and super-organizing maps in R: the kohonen package. J. Stat. Software 21, 1–19. 10.18637/jss.v021.i05

[B67] Wei L. Q. Xu W. Y. Deng Z. Y. Su Z. Xue Y. Wang T. (2010). Genome-scale analysis and comparison of gene expression profiles in developing and germinated pollen in *Oryza sativa* . BMC Genomics 11, 338. 10.1186/1471-2164-11-338 20507633 PMC2895629

[B68] Xiong Y. Contento A. L. Bassham D. C. (2007a). Disruption of autophagy results in constitutive oxidative stress in *Arabidopsis* . Autophagy 3, 257–258. 10.4161/auto.3847 17312382

[B69] Xiong Y. Contento A. L. Nguyen P. Q. Bassham D. C. (2007b). Degradation of oxidized proteins by autophagy during oxidative stress in *Arabidopsis* . Plant Physiol. 143, 291–299. 10.1104/pp.106.092106 17098847 PMC1761971

[B70] Xu J. Ding Z. Vizcay-Barrena G. Shi J. X. Liang W. Q. Yuan Z. (2014). *ABORTED MICROSPORES* acts as a master regulator of pollen wall formation in *Arabidopsis* . Plant Cell 26, 1544–1556. 10.1105/tpc.114.122986 24781116 PMC4036570

[B71] Yang C. Vizcay-Barrena G. Conner K. Wilson Z. A. (2007). MALE STERILITY1 is required for tapetal development and pollen wall biosynthesis. Plant Cell 19, 3530–3548. 10.1105/tpc.107.054981 18032629 PMC2174882

[B72] Yang Z. Liu L. Sun L. Yu P. Zhang P. Abbas A. (2019). OsMS1 functions as a transcriptional activator to regulate programmed tapetum development and pollen exine formation in rice. Plant Mol. Biol. 99, 175–191. 10.1007/s11103-018-0811-0 30610522

[B73] Yoshimoto K. Ohsumi Y. (2018). Unveiling the molecular mechanisms of plant autophagy-from autophagosomes to vacuoles in plants. Plant Cell Physiol. 59, 1337–1344. 10.1093/pcp/pcy112 29893925

[B74] Yoshimoto K. Jikumaru Y. Kamiya Y. Kusano M. Consonni C. Panstruga R. (2009). Autophagy negatively regulates cell death by controlling NPR1-dependent salicylic acid signaling during senescence and the innate immune response in *Arabidopsis* . Plant Cell 21, 2914–2927. 10.1105/tpc.109.068635 19773385 PMC2768913

[B75] Yoshimoto K. Shibata M. Kondo M. Oikawa K. Sato M. Toyooka K. (2014). Organ specific quality control of plant peroxisomes is mediated by autophagy. J. Cell Sci. 127, 1161–1168. 10.1242/jcs.139709 24463818

[B76] Yoshimoto K. (2012). Beginning to understand autophagy, an intracellular self-degradation system in plants. Plant Cell Physiol. 53, 1355–1365. 10.1093/pcp/pcs099 22764279

[B77] Yu J. Zhen X. Li X. Li N. Xu F. (2019). Increased autophagy of rice can increase yield and nitrogen use efficiency (NUE). Front. Plant Sci. 10, 584. 10.3389/fpls.2019.00584 31134120 PMC6514234

[B78] Zhang D. Yang L. (2014). Specification of tapetum and microsporocyte cells within the anther. Curr. Opin. Plant Biol. 17, 49–55. 10.1016/j.pbi.2013.11.001 24507494

[B79] Zhang W. Sun Y. J. Timofejeva L. Chen C. B. Grossniklaus U. Ma H. (2006). Regulation of *Arabidopsis* tapetum development and function by *dysfunctional tapetum1* (*dyt1*) encoding a putative bHLH transcription factor. Development 133, 3085–3095. 10.1242/dev.02463 16831835

[B80] Zhang Z. B. Zhu J. Gao J. F. Wang C. Li H. Li H. (2007). Transcription factor AtMYB103 is required for anther development by regulating tapetum development, callose dissolution and exine formation in *Arabidopsis* . Plant J. 52, 528–538. 10.1111/j.1365-313X.2007.03254.x 17727613

[B81] Zhang D. S. Liang W. Q. Yuan Z. Li N. Shi J. Wang J. (2008). Tapetum degeneration retardation is critical for aliphatic metabolism and gene regulation during rice pollen development. Mol. Plant 1, 599–610. 10.1093/mp/ssn028 19825565

[B82] Zhang D. Luo X. Zhu L. (2011). Cytological analysis and genetic control of rice anther development. J. Genet. Genomics 38, 379–390. 10.1016/j.jgg.2011.08.001 21930097

[B83] Zhen X. Xu F. Zhang W. Li N. Li X. (2019). Overexpression of rice gene *OsATG8b* confers tolerance to nitrogen starvation and increases yield and nitrogen use efficiency (NUE) in *Arabidopsis* . PloS One 14, e0223011. 10.1371/journal.pone.0223011 31553788 PMC6760796

[B84] Zhu E. G. You C. J. Wang S. S. Cui J. Niu B. X. Wang Y. X. (2015). The DYT1-interacting proteins bHLH010, bHLH089 and bHLH091 are redundantly required for *Arabidopsis* anther development and transcriptome. Plant J. 83, 976–990. 10.1111/tpj.12942 26216374

