# Supplementary material for: Impact of Autophagy on Gene Expression and Tapetal Programmed Cell Death During Pollen Development in Rice
Source: Front Plant Sci. 2020 Mar 6;11:172. doi: 10.3389/fpls.2020.00172 (PMC7068715; doi:10.3389/fpls.2020.00172)
Supplement: Supplementary file 6 [file Presentation_1.pdf]

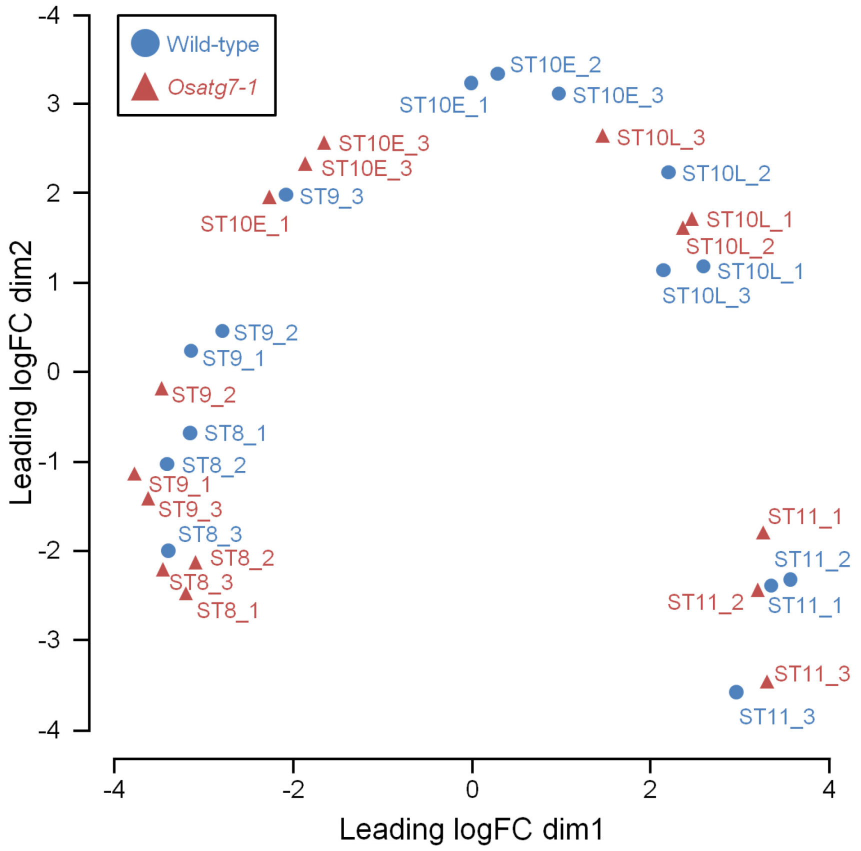

Supplementary Figure 1. MDS plot analyses. MDS was performed via calculating log-fold changes between wild-type and using ASDs to compute distances in EdgeR with the "plotMDS" function.

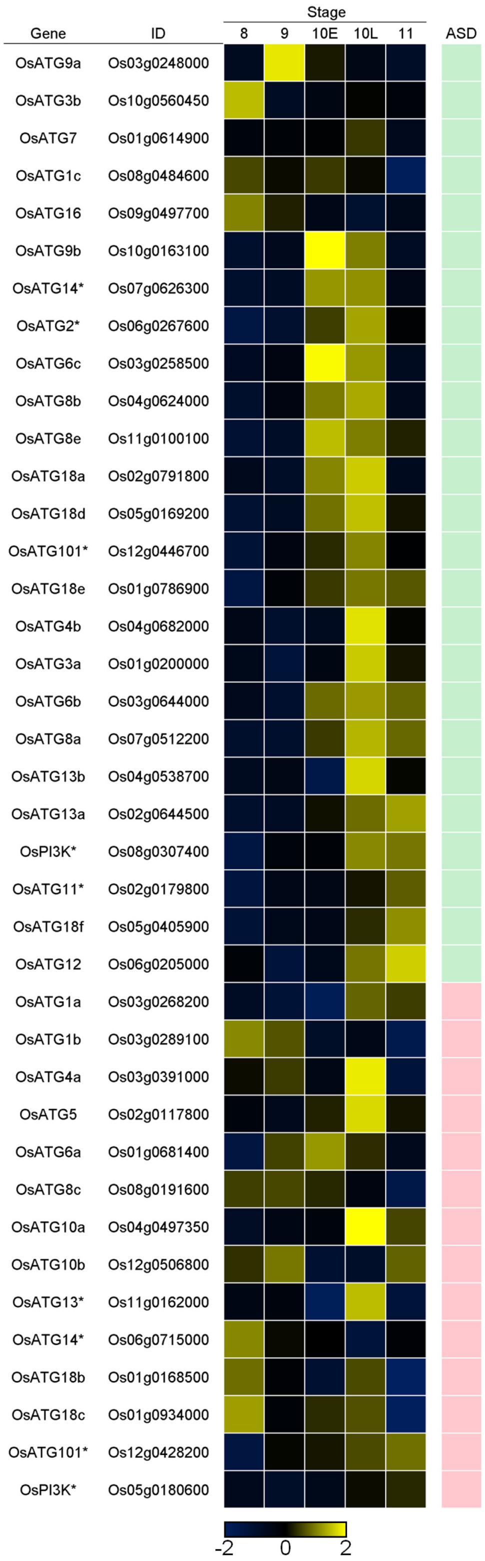

Supplementary Figure 2. Expression profiles of all *ATG* genes of rice anthers during pollen development. The heatmap shows in scaled expression patterns of *ATG* genes of rice during anther developmental stages in wild-type anthers. Green and Pink boxes indicate ASD (FDR < 0.01) or not (FDR > 0.01), respectively.

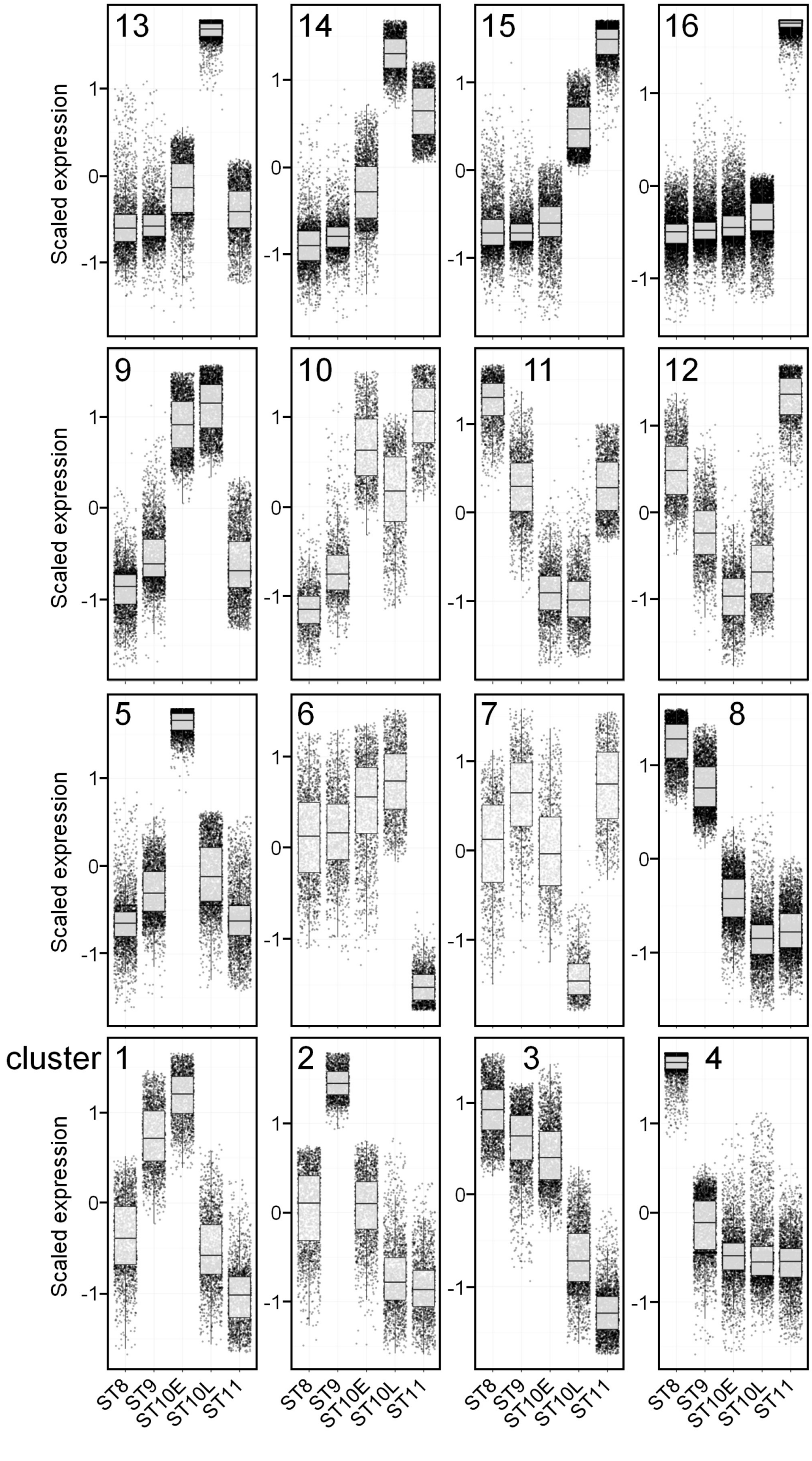

Supplementary Figure 3. Box plots showing expression patterns of each cluster. Scaled expression between the wild-type and *Osatg7-1* mutant plotted during 10 different conditions (wild-type and *Osatg7-1*, 5 developmental stages) is shown. Box plot explanation: upper horizontal line of box, 75th percentile; lower horizontal line of box, 25th percentile; horizontal bar within box, median; upper horizontal bar outside box, 90th percentile; lower horizontal bar outside box, 10th percentile.

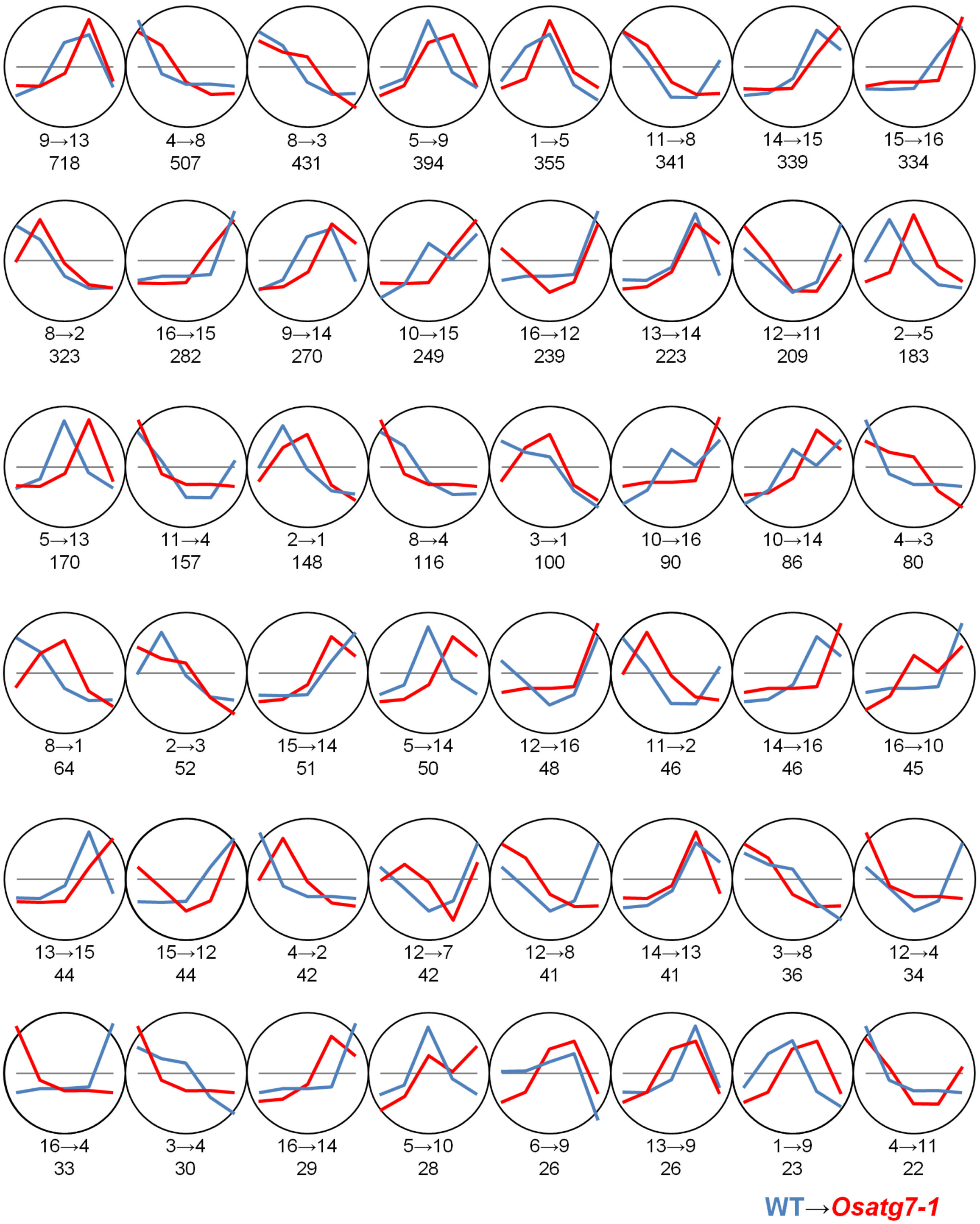

Supplementary Figure 4. Displacement directions after SOM clustering of data that were scaled separately from wild-type to *Osatg7-1*. Line plots indicate representative expression patterns in each cluster.

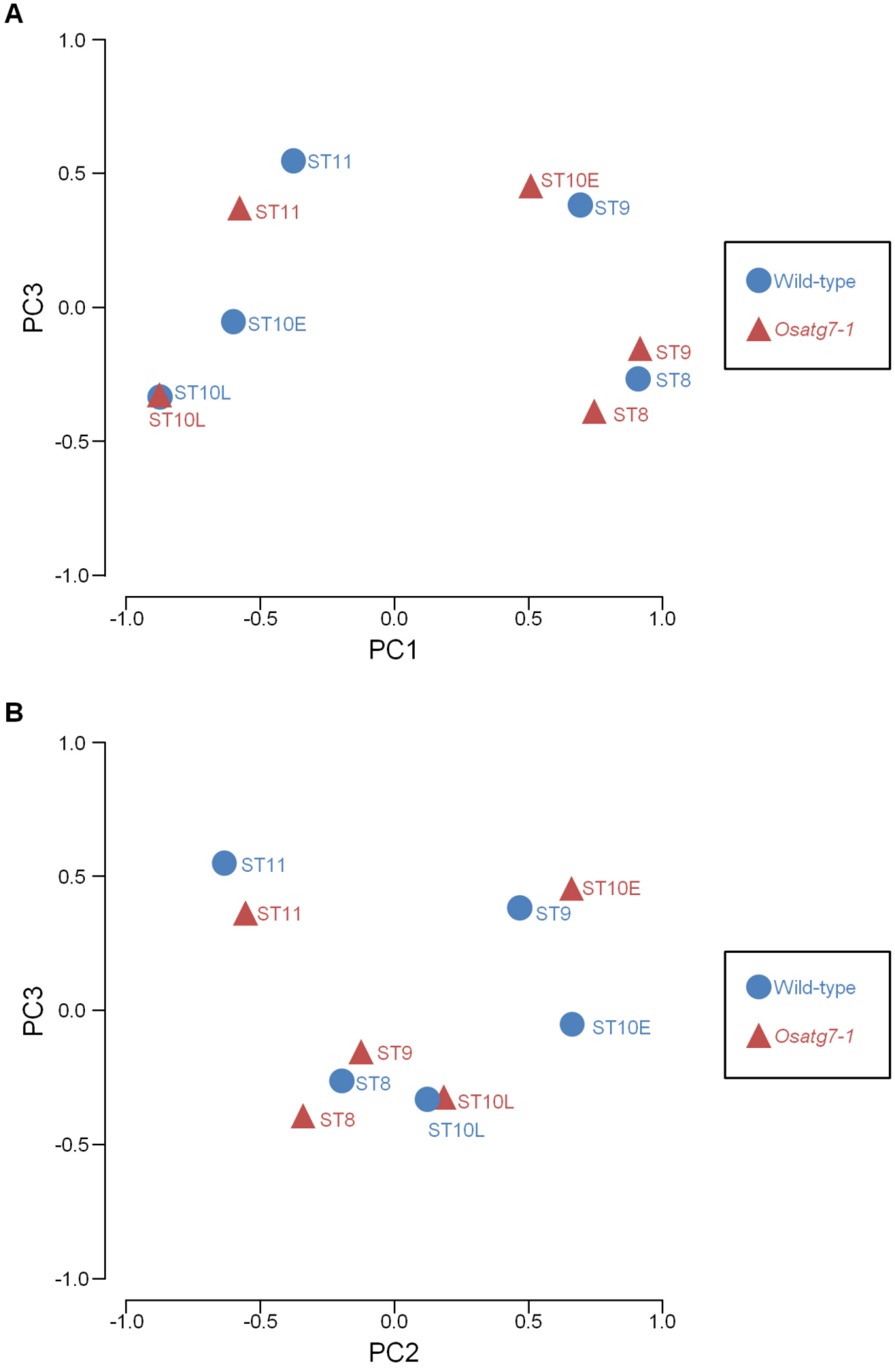

Supplementary Figure 5. PCA of gene expression. The global expression profile of each transcript is represented as PC1 and PC3 (A) and PC2 and PC3 (B). Note distinct dissimilarities between the wild-type and *Osatg7-1* mutant at ST9 and ST10E.

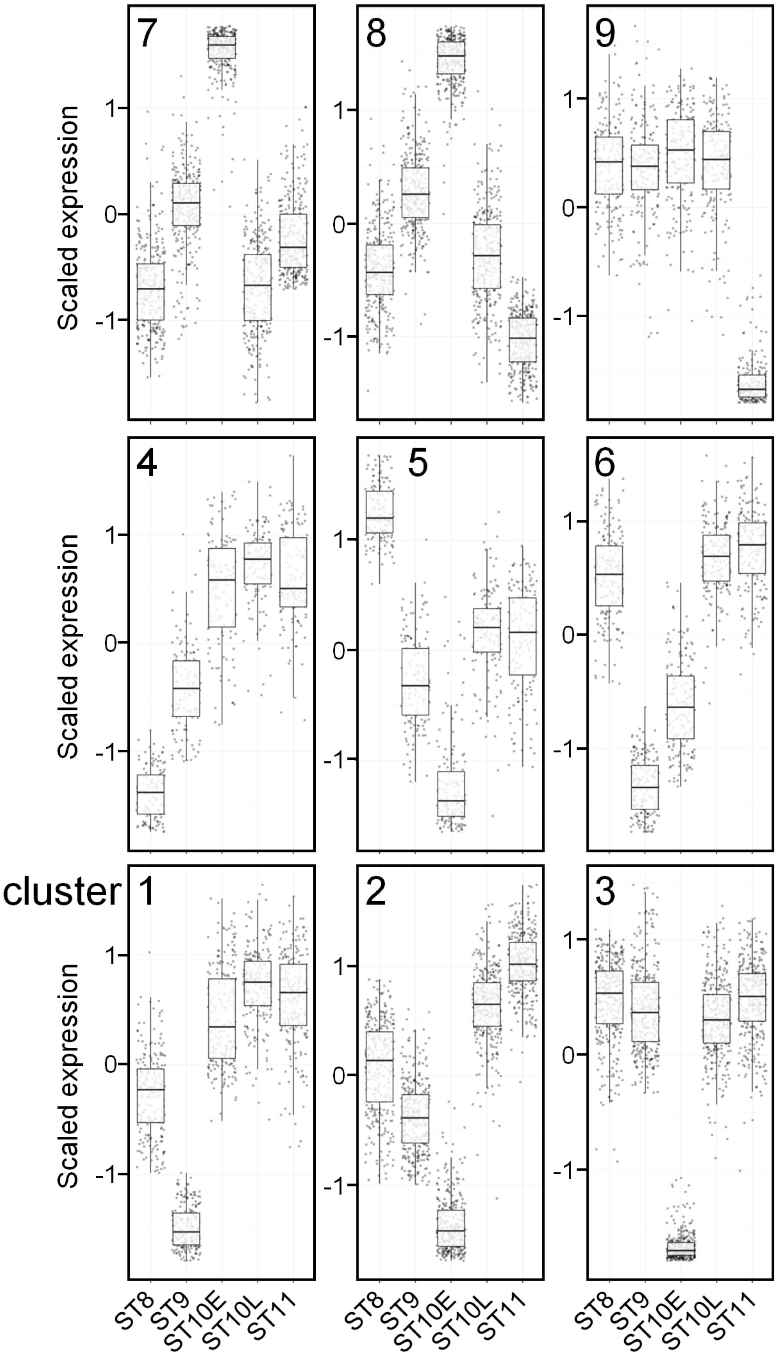

Supplementary Figure 6. Box plots showing expression patterns of each cluster. Scaled expression patterns between the wild-type and *Osatg7-1* mutant plotted at ST8, ST9, ST10E, ST10L, and ST11 are shown. Box plot explanation: upper horizontal line of box, 75th percentile; lower horizontal line of box, 25th percentile; horizontal bar within box, median; upper horizontal bar outside box, 90th percentile; lower horizontal bar outside box, 10th percentile.

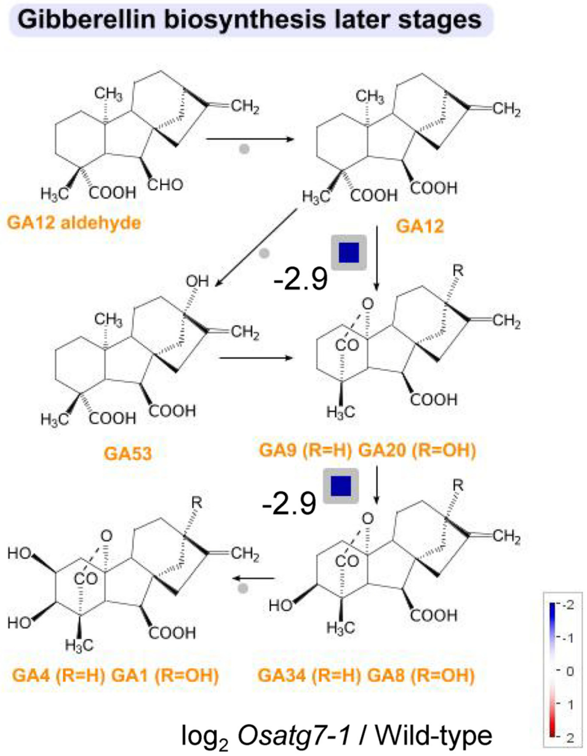

Supplementary Figure 7. Overview diagram of associated with ADDs at stage 10E in GA-biosynthesis later stages by MapMan analysis. The color key represents normalized log2 values. Red represents up-regulation and blue represents down- regulation between the wild-type and *Osatg7-1* mutant.

Supplemental Table S1. Primers used in this qPCR experiment.

Gene name	Accession No.	Primer sequence	Size (bp)
TIP2	Os01g0293100	F: GGCGACTGCCACATCTACA<br>R: ATCGGTCGGTCGGCTAA	140
EAT1	Os04g0599300	F: GAAGAAGGCCAACTCTCTGCT<br>R: GCATAAACCGCCGAACCT	144
GAMyb	Os01g0812000	F: ACTGAATCCACCCCTCCTGT<br>R: GCCCCATTACTTGCTCTCCA	109
AP38	Os04g0596200	F: ATGCCGATGCTGGTGGA<br>R: GTAGAGGCTTGGATGCGTGAG	132
AP25	Os03g0186900	F: GAACATCCGGGTCGTCTTC<br>R: AAGCCGTTCTCGTGCATTT	150
CYP704B2	Os03g0168600	F: GTTGAGATCGGGACGCTGT<br>R: CTCGATGAACCGCGACAGTA	279
MYB80	Os04g0470600	F: GAATAGTTTTACCGAGAAGCACTGA<br>R: CGACTGAAGCAGAGGACTGG	82
C4	Os08g0546300	F: CATGAACAGCCTGCCTTCC<br>R: CAGCAACCGGGCATTACA	233
UBQ5	Os01g0328400	F: ACCACTTCGACCGCCACTACT<br>R: ACGCCTAAGCCTGCTGGTT	69
